# A Comprehensive Review of Optical Metrology and Perception Technologies

**DOI:** 10.3390/s25226811

**Published:** 2025-11-07

**Authors:** Shuonan Shan, Fangyuan Zhao, Zinan Li, Linbin Luo, Xinghui Li

**Affiliations:** Tsinghua Shenzhen International Graduate School, Tsinghua University, Shenzhen 518055, China; shansn24@mails.tsinghua.edu.cn (S.S.); fy-zhao24@mails.tsinghua.edu.cn (F.Z.); lizn23@mails.tsinghua.edu.cn (Z.L.); luolb24@mails.tsinghua.edu.cn (L.L.)

**Keywords:** optical metrology, perception technology, interferometry, imaging, spectroscopy, hybrid optical systems, multimodal sensing, cross-domain integration, precision measurement

## Abstract

Optical metrology and perception technologies employ light as an information carrier to enable non-contact, high-precision measurement of geometry, dynamics, and material properties. They are widely deployed in industrial and consumer domains, from nanoscale defect inspection in semiconductor manufacturing to environmental perception in autonomous driving and spatial tracking in AR/VR. However, existing reviews often treat individual modalities—such as interferometry, imaging, or spectroscopy—in isolation, overlooking the increasing cross-domain integration in emerging systems. This review proposes a hierarchical taxonomy encompassing four core systems: interferometry, imaging, spectroscopy, and hybrid/advanced methods. It introduces a “theory–application–innovation” framework to unify fundamental principles, application scenarios, and evolutionary trends, revealing synergies across modalities. By mapping technological progress to industrial and societal needs, including AI-driven optimization and quantum-enhanced sensing, this work provides a structured, evolving knowledge base. The framework supports both cross-disciplinary understanding and strategic decision-making, offering researchers and engineers a consolidated reference for navigating the rapidly expanding frontiers of optical metrology and perception.

## 1. Introduction

**Figure 1:**
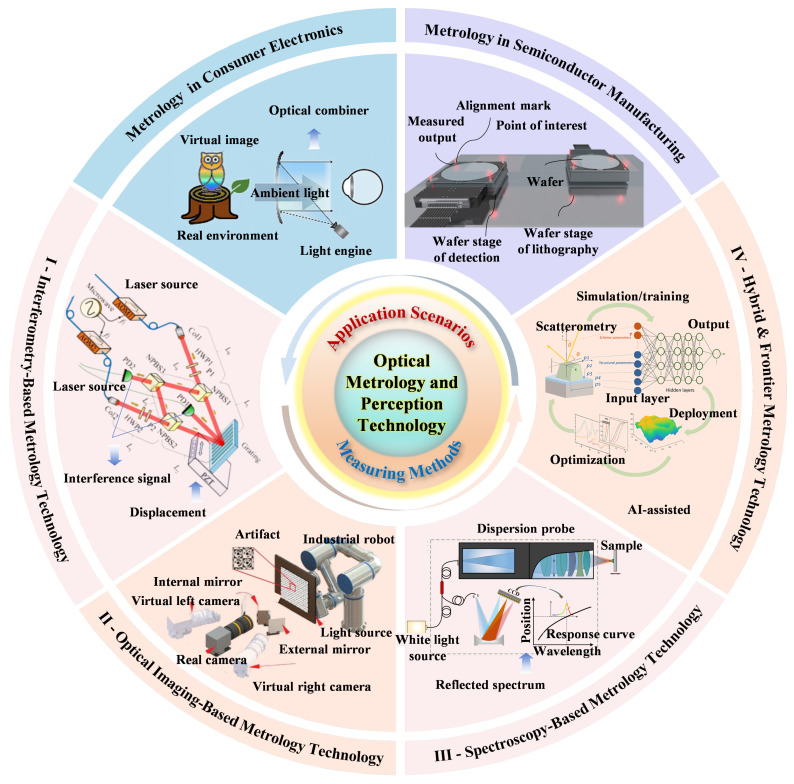
Overview of application scenarios and technology classification of optical metrology and perception.

Optical metrology and perception technologies employ light waves as carriers of information to enable precise quantification and perception of geometric morphology, dynamic behaviors, and material properties of target objects by measuring light intensity, phase, wavelength, polarization, and other physical parameters. Distinguished by their non-contact nature, high precision, and exceptional sensitivity, these technologies have become indispensable in both industrial and consumer domains. In industrial settings, they support critical processes such as nanoscale defect inspection in semiconductor manufacturing, high-precision positioning for robotic manipulation, and environmental perception for autonomous driving (e.g., LiDAR-based 3D reconstruction). On the consumer side, they underpin applications ranging from spatial interaction tracking in AR/VR to biometric authentication (e.g., facial recognition) and motion capture for immersive entertainment, as shown in Figure 1. As shown in Table 1, optical measurement techniques cover multiple spatial scales, ranging from nanometer-level interferometry to cross-scale computational imaging techniques. With the advancement of smart manufacturing [1,2] and the rise of immersive digital ecosystems, optical sensing is evolving toward greater multidimensionality, higher temporal resolution [3,4], and intelligent adaptability [5].

Despite substantial progress, existing literature tends to focus narrowly on specific technical branches, such as structured-light 3D imaging, interferometric displacement metrology, or spectral inspection, without sufficiently addressing the cross-domain synergies that increasingly define the field. Modern optical measurement systems often integrate multiple modalities [6]—interferometry leverages wave interference for nanometric displacement and surface roughness analysis [7]; imaging techniques span from triangulation-based contour scanning to computational imaging through scattering media; spectroscopy underpins both material identification and dimensional metrology; and hybrid/advanced methods, including fringe projection for deformation analysis, time-of-flight depth sensing, quantum-enhanced interferometry, and metasurface-enabled wavefront control—are pushing performance frontiers. Traditional taxonomies, however, struggle to accommodate such convergence—e.g., light-field imaging that blends geometric optics with computational reconstruction, or spectral confocal techniques that merge spectroscopic and spatial measurement principles.

Fundamentally, the objectives of optical measurement and perception can be distilled into two primary categories: positioning and surface contour characterization. Positioning encompasses precision motion control and accurate determination of spatial posture, while contour characterization involves capturing macroscopic shape, positional deviations, fine-scale profile, roughness, and other surface integrity parameters. These goals form the performance benchmarks by which diverse optical metrology techniques are evaluated, and they provide a common ground for integrating different modalities into coherent measurement strategies.

In this context, a comprehensive and integrative review is both timely and necessary. This work classifies optical metrology into four core systems—interferometry, imaging, spectroscopy, and hybrid or advanced methods—within a “theory–application–innovation” analytical framework. This structure reveals the links between different techniques, traces their evolution, and connects technological progress with industrial and societal needs such as AI-driven optical optimization and quantum-enhanced sensing. The review provides both a technical reference for researchers and a strategic guide for engineers and planners working in the expanding fields of optical metrology and perception.

In this review, the concept of optical perception is introduced as an evolutionary extension of optical metrology. While metrology primarily focuses on achieving quantitative precision in measurement—such as displacement, wavelength, or surface profiling—perception emphasizes the ability of optical systems to interpret, recognize, and respond to complex environmental information. Through the integration of computational algorithms, artificial intelligence, and data-driven modeling, modern optical systems are transitioning from traditional measurement instruments to perception-oriented platforms capable of contextual understanding and adaptive decision-making. In this framework, metrology provides the quantitative foundation, whereas perception represents the cognitive extension of optical measurement.

Scope note: The discussion focuses on industrial and consumer applications (e.g., manufacturing, robotics, automotive), excluding specialized domains such as Raman spectroscopy in materials science or biomedical imaging. While comprehensive coverage is inherently challenging amid rapid innovation, the intent is to offer a scalable framework that can be updated as the field advances.

## 2. Interferometry-Based Metrology

**Figure 2:**
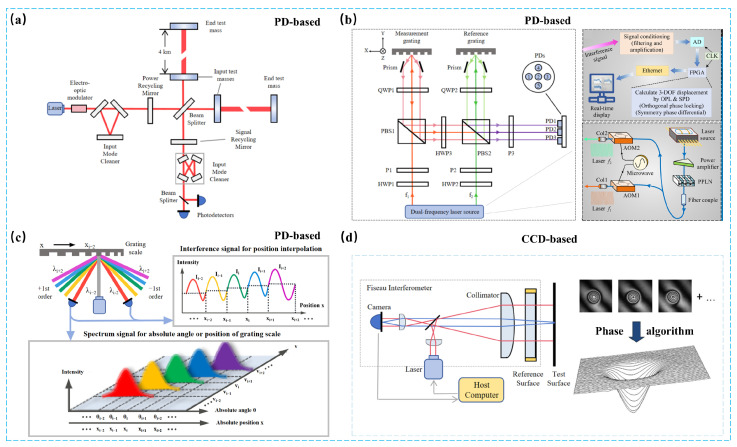
Interference-based measurement methods: (**a**) LIGO laser interferometer; (**b**) grating interferometer; (**c**) optical frequency comb; (**d**) Fizeau interferometer.

Optical interferometry-based metrology can be categorized into photodetector (PD)-based methods and CCD-based methods. PD-based interferometric systems rely on photodetectors to decode interference patterns and serve as the cornerstone of high-precision metrology [8]. Such approaches primarily encompass three techniques: grating interferometry, laser interferometry, and optical frequency comb interferometry. This section provides an review of their underlying principles, recent technological advances, and representative applications. Figure 2 shows the common interference-based measurement methods.

### 2.1. Laser Interferometry

Laser interferometry is a cornerstone of modern precision metrology. By utilizing the interference of coherent laser beams, it provides displacement, angle, and vibration measurements with exceptional resolution—ranging from nanometers down to picometers or even femtometers. Its flexibility in design and adaptability to various modulation techniques has led to numerous implementations tailored to specific application needs. Figure 2a shows the basic construction principle of the Laser Interferometer Gravitational-Wave Observatory (LIGO) system, which is based on laser interferometry technology. This section presents a detailed examination of several core configurations, including homodyne, heterodyne, and superheterodyne systems, along with specialized modalities and their use in advanced metrology.

#### 2.1.1. Homodyne Systems

A homodyne laser interferometer employs a single-frequency laser source and extracts phase information from the intensity variations of the interference signal. Let the reference arm field be $E_{r}\left(t\right)=A_{r}e^{i(\omega t+\phi_{r})}$, and the measurement arm field be $E_{s}\left(t\right)=A_{s}e^{i(\omega t+\phi_{s})}$. The resulting superimposed field leads to an intensity of

(1) $$I\left(t\right)=|E_{r}+E_{s}{|}^{2}=A_{r}^{2}+A_{s}^{2}+2A_{r}A_{s}\cos\left(\Delta \phi \right)$$

where $\Delta \phi =\phi_{s}-\phi_{r}$ is related to the optical path difference ΔL via $\Delta \phi =\frac{2\pi }{\lambda }\Delta L$. By measuring relative changes in phase, one can determine the corresponding displacement, deformation, vibration, and other quantities of interest.

Homodyne interferometry can achieve sub-nanometer precision, with a theoretical non-ambiguity range of λ/2. To extend this range, phase unwrapping techniques are required. A common approach to phase retrieval is to generate two orthogonal signals using polarization optics, from which the phase is calculated via an arctangent operation. The homodyne configuration is advantageous in its structural simplicity, low cost, and ease of integration. However, because the measurement signal contains a DC component, it is susceptible to DC drift induced by external disturbances, which degrades phase estimation accuracy. Furthermore, due to inherent imperfections in optical components and environmental influences, perfect orthogonality between signals is rarely achieved, introducing additional nonlinear errors.

To address these limitations, a variety of compensation techniques have been developed. For instance, optical path-folding designs can enhance resolution without increasing system dimensions, while quadrant-based signal acquisition can suppress channel crosstalk. Zhang et al. implemented a homodyne SPM system with real-time phase delay correction, achieving less than 1 nm error in large-range displacement scanning interferometry [9]. In practice, homodyne interferometers have been widely employed in scanning probe microscopy (SPM), nanoscale feedback loops, and angle encoders. Notably, both the Renishaw XL-80 laser system and the API 5D system are based on the homodyne configuration [10,11], and see extensive use in production environments for machine tool calibration and linear axis verification. Further advancements include adaptive gain-based phase compensation algorithms; for example, Keem et al. proposed a real-time gain adjustment method in which the preamplifier gains of a quadrant detector are tuned to effectively eliminate periodic errors caused by imperfections in the polarization beam splitter, achieving experimental errors as low as 0.04 nm [12].

#### 2.1.2. Heterodyne Interferometry

A heterodyne interferometric system employs two optical beams with a small frequency difference $\Delta f=f_{1}-f_{2}$ to generate a time-varying beat signal. Let the two optical fields be $E_{1}=A_{1}e^{i(\omega_{1}t+\phi_{1})}$, $E_{2}=A_{1}e^{i(\omega_{2}t+\phi_{2})}$, the resulting superimposed intensity can be expressed as

(2) $$I\left(t\right)=A_{1}^{2}+A_{2}^{2}+2A_{1}A_{2}\cos\left(\Delta \omega t+\Delta \phi \right)$$

where Δω=2πΔf and $\Delta \phi =\phi_{2}-\phi_{1}$ carries the information of the optical path difference. By processing the beat signal using a lock-in amplifier or a digital phase demodulator, Δϕ can be measured with high accuracy in the low-frequency domain. Compared with the amplitude-modulated signal in homodyne interferometry, the frequency-modulated signal in heterodyne interferometry inherently suppresses DC bias and exhibits greater robustness against environmental disturbances.

Heterodyne interferometric systems are particularly well-suited for long-range displacement measurements, as well as industrial applications requiring high dynamic range, immunity to zero drift, and precision measurements of displacement, vibration, or angular motion. With a stable frequency reference and high signal-to-noise ratio (SNR) detection, heterodyne interferometry often achieves relative measurement resolution from the picometer to sub-nanometer scale. Moreover, the beat frequency processing effectively down-converts the optical phase information into a frequency band more amenable to digital processing, thereby enhancing the stability of phase extraction.

For example, Joo et al. demonstrated a high-resolution heterodyne laser interferometer for linear displacement measurement that exhibited no periodic nonlinearity. Their design employed acousto-optic frequency shifting and spatial separation of the signal paths, thereby minimizing mode coupling and crosstalk [13,14]. Additional examples include Leirset’s GHz-range vibration sensor with femtometer sensitivity [15], Hao Yan’s dual-beam heterodyne sensor achieving 1 pm/Hz performance for linear and angular displacement [16], and Dong’s heterodyne system using phase-locked loop demodulation to reach 10-pm resolution in both air and vacuum environments [17]. These advances highlight the adaptability of heterodyne methods in emerging micro/nano-motion sensing applications.

#### 2.1.3. Superheterodyne Interferometry

Optical super-heterodyne interferometry draws inspiration from the super-heterodyne concept in radio technology: two (or more) optical frequencies are mixed, either electronically or optically, to generate an intermediate frequency (IF) that can be directly measured. This enables the detection of phase or frequency differences that are difficult to measure at the original frequency separation (for example, when the frequency difference between two optical waves exceeds the bandwidth of the electronic detector).

For a dual-wavelength/dual-frequency source $\lambda_{1}$ and $\lambda_{2}$, if each is mixed with a local oscillator (or a modulated local oscillator), a low-frequency IF can be obtained, whose phase contains the phase difference information of the two beams. Super-heterodyne techniques are often used to generate a “synthetic wavelength” Λ to increase the non-ambiguity range. The synthetic wavelength Λ satisfies:

(3) $$\Lambda =\frac{\lambda_{1}\lambda_{2}}{|\lambda_{1}-\lambda_{2}|}$$

and provides displacement information over a larger range via the synthetic phase $\Phi_{\Lambda }$. Implementation methods for super-heterodyne detection include AOM/EO modulation, electronic down-conversion, and optoelectronic mixing of frequency combs or swept sources. In a two-wavelength super-heterodyne system, the use of the synthetic wavelength allows the phase measurement to be converted into a measurement corresponding to a longer “equivalent wavelength,” thereby extending the non-ambiguity range while maintaining high phase sensitivity. However, the synthetic wavelength is inherently sensitive to noise, wavelength drift, and system calibration, and the ultimate absolute measurement uncertainty is determined by the relative wavelength accuracy of each monochromatic source and the system phase noise. In practical applications, absolute ranging accuracy from sub-millimeter to sub-micrometer can be achieved, making this approach suitable for scenarios requiring either a large measurement range or absolute distance measurement.

Le Floch et al. proposed a novel super-heterodyne technique based on the two-wavelength interferometry (TWI) method for long-distance measurement, in which the two frequencies are generated from synchronously scanned optical and radio frequencies, achieving an accuracy of ±50 μm over a range of 1–15 m [18]. Yin et al. combined heterodyne and super-heterodyne interferometers to realize simultaneous multi-wavelength detection and demodulation, achieving an accuracy of 17 μm within a 2 m range in their experiments [19].

#### 2.1.4. Specialty Modalities

Beyond the classical homodyne and heterodyne architectures, several specialized laser interferometry techniques have emerged to serve application-specific requirements:

Fabry–Perot Interferometers (FPI) have shown excellent sensitivity and compactness for refractive index, gas pressure, and temperature sensing. Liu and Qu fabricated an FPI cavity via femtosecond laser-induced water breakdown and arc annealing, achieving high fringe visibility (30 dB), sensitivity (1147.48 nm/RIU), and temperature insensitivity, suitable for liquid refractive index measurements [20]. Xu et al. developed a fiber FPI with a glass microsphere enabling simultaneous gas pressure and temperature sensing via multi-beam interference, offering robust, easy-to-fabricate devices for industrial monitoring [21].

Self-Mixing Interferometry (SMI) uses feedback light re-entering the laser cavity to modulate output intensity in a compact setup. Albert et al. developed a theoretical and experimental framework for long-distance (>10 m) SMI, demonstrating practical vibration and displacement sensing with SNR = 3.6 at 12 m [22]. Zhang et al. introduced a multichannel SMI system employing injection current modulation and frequency division multiplexing, achieving multi-target displacement sensing with <1.4% relative error. These advances extend SMI to distributed sensing and precision manufacturing [23].

Dual-Comb Interferometry (DCI) exploits two stabilized frequency combs for fast, absolute distance and spectral measurements without mechanical scanning. Herman et al. achieved picometer-level displacement precision via carrier-envelope phase tracking [24]. Deng et al. demonstrated a high-coherence dual-comb system enabling simultaneous gas spectroscopy and absolute distance measurement with 0.68 μm precision and 40 μm lateral resolution. DCI excels in speed, spectral resolution, and multifunctionality, ideal for environmental and aerospace metrology [25].

Frequency-Sweeping Interferometry (FSI) encodes distance via tunable laser wavelength sweeping for large-range absolute measurements with simple hardware. Zhang et al. improved FSI accuracy and stability at 4.5 m by integrating reference interferometers and target drift compensation [26]. Coggrave et al. addressed high sampling demands in long-range FSI using silicon photonic adaptive delay lines, enabling chip-scale integration. FSI offers cost-effective, flexible solutions but requires precise wavelength calibration and drift control for high accuracy [27].

### 2.2. Grating Interferometry

Grating interferometry employs the interference between diffracted beams from periodic structures to achieve displacement measurement with sub-nanometer resolution [28,29,30]. Due to its modular design, compact structure [31,32,33], and high degree-of-freedom (DOF) expandability [34], it has been increasingly adopted in semiconductor lithography [35,36,37], ultra-precision manufacturing [38,39,40], and optical encoder systems [41,42,43,44,45].

#### 2.2.1. Single-DOF and Planar Systems

Initial implementations of grating interferometry were primarily focused on 1-DOF displacement measurement. By combining ±1st-order diffracted beams from a reflective or transmissive grating, phase shifts induced by linear displacement could be accurately resolved [46,47,48,49]. Commercial systems such as Magnescale’s laser encoders reach a resolution of 0.017 nm using small-period gratings and optical subdivision techniques. To enhance measurement stability and alignment tolerance, designs have evolved to include self-collimating incidence and quasi-common-path configurations. Hsieh et al. [50] proposed a quasi-common-path heterodyne system that achieved sub-3 nm resolution under practical operation conditions. Wu et al. [51] developed a Littrow configuration grating interferometer that expands Z-directional range and maintains high contrast fringe quality, making it well-suited for vertical stage calibration. For planar (2D) systems, Kimura et al. [52,53,54] constructed a reflective scale grating encoder capable of simultaneous X–Z detection with sub-nanometer resolution, particularly useful for high-speed precision stages. Yin et al. [55] and Yang et al. [56] designed heterodyne double-spatial systems and fiber-coupled grating interferometers for XY displacement tracking. Their systems achieved in-plane stability of 0.246 nm and out-of-plane resolution of 0.465 nm, even under environmental drift.

#### 2.2.2. Three-DOF and Six-DOF Systems

As metrology demands increase for wafer stages, air-bearing platforms, and robotic motion systems, grating interferometry has expanded into multi-dimensional configurations [57,58,59]. These systems not only track translational motion (X, Y, Z) but also rotational and angular displacements (pitch, yaw, roll) [60]. Compact 3-DOF systems have been realized using pyramid-prism beam shaping and planar cross-gratings [61]. Wang [62] et al. demonstrated a compact 3D encoder with better than 500 nm accuracy across three axes. Kimura et al. introduced a sub-nanometer resolution three-axis surface encoder using a hybrid reflective planar grating and collimated sensing beam.

For full 6-DOF tracking, Hsieh et al. [63] proposed a Michelson-grating hybrid interferometer integrating grating shearing and heterodyne detection. The system achieved 2 nm displacement resolution and a 0.05 μrad angular accuracy. Lin et al. improved the optical diffraction efficiency and SNR of 2D/3D configurations using gold-coated cross gratings with theoretical signal contrast near 100%. Cui et al. [64] report an integrated zero-dead-zone heterodyne grating interferometer that enables simultaneous 3-DOF atomic-level displacement measurement. Utilizing a dual-frequency laser and a symmetrical optical path, the system eliminates dead-zone error and achieves sub-nanometer resolution (0.25 nm in X/Y, 0.3 nm in Z), high linearity ($10^{-5}$), and 0.8 nm repeatability, as shown in Figure 2b. The design significantly improves precision and environmental stability in multi-axis metrology. These developments are critical for next-generation photolithography systems [65,66] and high-precision robotic metrology [67,68], where six-dimensional feedback is a necessity.

#### 2.2.3. Multi-Optical-Head Architectures

To expand spatial measurement volume and address complex motion control systems, multi-head grating interferometers are implemented. These systems consist of several spatially distributed optical heads referencing a single grating target or surface, enabling comprehensive rigid-body pose reconstruction [69,70,71,72]. One prominent industrial example is ASML’s 6-DOF metrology platform, which employs multiple grating sensors across a wafer stage. The redundancy and spatial diversity of measurements enable high accuracy (sub-micron linear, sub-arcsecond angular) across meter-scale motion envelopes. Recent multi-head implementations also integrate real-time thermal compensation [73], modular readout electronics [74], and parallel FPGA-based signal processing [75], extending their use in precision gantry systems, coordinate measuring machines (CMMs) [76], and space-constrained environments such as in-line inspection arms.

While grating interferometers offer numerous advantages, they are not without challenges [77,78]. Common issues include phase errors from grating non-uniformity [79,80,81], alignment sensitivity [82], thermal expansion of scale substrates, and difficulty in demodulating multi-DOF interference signals with high fidelity [83,84,85]. Emerging solutions focus on improved grating fabrication via nanoimprint lithography or interference lithography [86,87,88,89,90,91,92], error modeling using phase chain compensation, and signal enhancement through machine learning-assisted demodulation algorithms [93]. There is also increasing interest in integrating smart sensors and edge-AI modules directly into encoder heads to support adaptive correction and in-situ diagnostics.

In particular, grating parameters significantly affect the accuracy and resolution of grating interferometers [94,95,96,97,98]. Cheng Xinbin et al. [99] proposed an atomic lithography technology, through which one-dimensional and two-dimensional chromium self-traceable gratings are prepared. The grating pitch is directly traced back to the chromium atomic transition frequency (natural constant), and the pitch accuracy reaches the picometer level (0.001 nm), which may provide a new solution for further grating interferometry.

### 2.3. Optical Frequency Comb-Based Interferometry

Optical frequency combs (OFCs), consisting of a series of equally spaced, phase-coherent optical modes, have revolutionized the field of precision measurement [100,101]. When coupled with photodetector-based interferometric techniques, OFCs enable absolute, high-precision displacement and distance measurements with remarkable resolution and traceability to primary time and length standards. Their intrinsic frequency stability and ultra-broadband spectral characteristics allow for applications that are challenging or inaccessible to traditional single-wavelength interferometers [102,103].

Optical frequency combs (OFCs) have significantly advanced precision measurement techniques by offering a highly stable and spectrally coherent reference composed of evenly spaced optical frequencies. Unlike traditional interferometry that typically employs a single frequency or a limited set of discrete wavelengths, frequency comb interferometry uses hundreds or thousands of frequency lines simultaneously, each precisely defined and traceable to fundamental atomic standards. The fundamental principle involves interfering comb pulses or comb lines after they have traveled different optical paths. Photodetectors then convert this optical interference into electrical signals containing information about the relative optical delays, directly translating into absolute displacement or distance measurements [7,104].

#### 2.3.1. Absolute Distance Measurement

The narrow linewidth, evenly spaced comb-tooth structure of an optical frequency comb, together with its direct traceability to radio-frequency (RF) or optical standards, makes it an important light source for high-precision absolute distance measurement. In early work, Minoshima and Matsumoto [105] employed a train of femtosecond laser pulses as a time ruler to implement a time-of-flight (TOF)-based absolute ranging method, achieving an accuracy of 3 μm over a range of 240 m, and improving real-time performance via multi-channel synchronization. Subsequently, Coddington et al. [106] proposed the dual-comb interferometry (DCI) method, in which two Er-doped fiber optical frequency combs were employed with a repetition rate difference of approximately 1 kHz, both locked to the same frequency standard. By generating an equivalent low-frequency interference signal through beat detection, mechanical scanning was eliminated, enabling ranging accuracy better than 1 μm over distances from 1 mm to 1 km. Lee et al. [107] combined an optical frequency comb with a tunable narrow-linewidth laser to generate multiple synthetic wavelengths, effectively extending the non-ambiguity range and achieving sub-micrometer accuracy over a measuring range of 100 m.

#### 2.3.2. Dynamic Measurement and High-Speed Profiling

In dynamic measurement and high-speed profilometry, dual-comb interferometry offers a promising approach for non-contact measurements with high sampling rates and high precision. Ideguchi et al. [108] proposed a time-stretch dual-comb interferometry technique, utilizing two optical frequency combs with a stable repetition rate difference to acquire interference signals from the surface of an object in real time. This enabled contour measurement at a rate of 25 kHz, applicable to monitoring high-speed vibrations or dynamic processes. Kobayashi et al. [109] built a dual-comb system capable of position tracking with sub-micrometer resolution for rapidly moving targets at a sampling rate of 500 Hz, suitable for online monitoring in precision manufacturing processes. In addition, Hase et al. [110] combined dual-comb interferometry with optical coherence tomography (OCT), achieving a high-speed tomographic measurement at a 1.2 MHz A-scan rate based on an Er-doped fiber comb at 1.55 μm. This setup simultaneously acquired surface and internal depth information of a structure, providing an effective solution for rapid three-dimensional imaging of complex structures.

Despite their advantages, OFC-based interferometric systems face technical challenges that currently restrict widespread adoption, particularly in industrial settings. Among these challenges, the complexity associated with stabilizing key comb parameters remains significant. Achieving the necessary frequency stability typically requires complex stabilization schemes involving precision electronic control, ultra-stable laser cavities, and optical frequency references, which increases both cost and system complexity.

### 2.4. CCD-Based Optical Interferometry

CCD-based precision measurement techniques utilize charge-coupled device (CCD) image sensors to directly record and analyze interference fringes, diffraction patterns, or holographic wavefronts, offering substantial advantages in spatial resolution, dynamic measurement capability, and non-contact surface characterization. In contrast to PD-based methods that primarily rely on single-point or limited-channel detection, CCD-based methods inherently provide full-field measurements, enabling simultaneous capture of extensive surface or volumetric information. Prominent examples within this category include white-light Fizeau interferometry and digital holographic microscopy (DHM), each characterized by distinct measurement principles and application contexts. This section comprehensively reviews the principles, recent technological advances, and representative applications of these two critical CCD-based precision measurement methods.

#### 2.4.1. Fizeau Interferometry

In the field of precision optical metrology, the Fizeau interferometer, invented by Hippolyte Fizeau in 1862, has become a cornerstone measurement tool owing to its common-path configuration, simplified optical alignment, and high stability [111,112]. The fundamental principle, as illustrated, involves splitting a light beam emitted from a source to illuminate both a reference surface and the test specimen. The reflected beams interfere, producing fringe patterns from which the surface morphology and minute deformations of the specimen can be precisely extracted through phase analysis.

To overcome mechanical limitations inherent in traditional phase-shifting techniques, Gary Sommargren proposed Wavelength Tuned Phase Shifting Interferometry (WPSI), wherein phase shifts are induced by tuning the laser wavelength [113]. The amount of phase shift is directly related to the number of reflections, optical thickness of the test sample, and frequency offset of the laser source, enabling enhanced precision and stability in measurements of large-aperture and complex optical components. For instance, at the United States National Ignition Facility (NIF), a 600 mm aperture WPSI Fizeau interferometer was employed to measure large laser amplifier plates sized 460 × 810 mm. Utilizing a Littman–Metcalf configuration laser enabling mode-hop-free wavelength tuning combined with cavity locking techniques to suppress vibration-induced errors, the system successfully fulfilled the stringent requirements for high-precision, large-scale optical metrology. This implementation demonstrates the significant potential and broad applicability of WPSI in large-aperture, high-accuracy optical measurements [114,115].

Recent advances have addressed longstanding challenges in Fizeau interferometry related to phase-shift accuracy, imaging errors, noise floor reduction, and measurement range extension. Xu et al. introduced a dual-stage correction method for phase shifters, combining an ultra-high linearity phase shifter with an auto velocity iterative correction, achieving nanometer-level displacement accuracy and sub-0.1% nonlinearity, thus significantly improving phase-shifting interferometry (PSI) precision [116]. Complementing this, Morrow et al. developed an empirical model to correct retrace and system imaging errors inherent in non-null Fizeau measurements of aspheric and curved optics, demonstrating sub-2 nm RMS accuracy for full-aperture, single-shot measurements critical in X-ray mirror fabrication [117].

Noise suppression has seen notable breakthroughs through anisotropic spatial-coherence engineering, as demonstrated by Li et al., who utilized illumination modulation to reduce mid-spatial-frequency noise below the sub-nanometer level, thereby enhancing sensitivity and measurement fidelity [118]. This methodological innovation provides a rapid, full-aperture solution for detecting subtle surface errors on high-precision optics.

Integration of novel beam structures has also advanced the field. Lu et al. explored the use of orbital angular momentum (OAM) beams within Fizeau interferometers. Their common-path OAM interferometric schemes combined with azimuthal phase demodulation enabled stable, compact measurement systems capable of resolving displacements down to tens of picometers without requiring traditional phase-shifting devices, thus offering robust and efficient phase retrieval methods [119,120].

From an instrumentation perspective, Kühnel et al. demonstrated a scanning differential interferometer-based profilometer capable of sub-nanometer 3D topography measurements on freeform optics with steep local slopes (up to 7 mrad), supporting large apertures up to 100 × 100 mm^2^. The system’s precision and repeatability were validated on silicon mirrors, confirming its suitability for advanced optical manufacturing [121].

Furthermore, Da Silva et al. developed a Fizeau interferometry stitching system to characterize large X-ray mirrors with sub-nanometer height errors [122]. By combining multiple overlapping sub-aperture scans using advanced stitching algorithms, the system achieved exceptional spatial resolution and reproducibility, critical for synchrotron and free electron laser applications requiring ultra-precise surface metrology.

The application scope of Fizeau interferometry has been considerably expanded in recent years, particularly in large-area precision manufacturing contexts. For instance, Zygo’s MST series employs an array of synchronized CCD-based interferometric sensors, enabling simultaneous high-resolution measurement of large optical components, such as telescope mirror segments [123]. Similarly, Taylor Hobson developed a large-area CCD-based profiler capable of rapidly inspecting precision-engineered surfaces, achieving nanometric resolutions over extensive surface areas [124].

Fizeau interferometry’s capability to measure complex surfaces with nanometric precision, combined with emerging techniques in noise suppression and phase demodulation, solidify its position in optical manufacturing, X-ray optics characterization, and freeform surface metrology. Remaining challenges involve further integration of correction algorithms, expansion of measurement ranges, and reduction of system complexity to facilitate wider industrial adoption.

#### 2.4.2. Digital Holographic Interferometry

Digital Holographic Interferometry (DHI) is a non-contact optical method that integrates holographic recording with interferometric measurement. Its principle involves forming interference fringes on the detector plane by the superposition of the object wave and the reference wave, capturing these fringes with a digital sensor (such as a CCD/CMOS), and reconstructing the complex amplitude distribution of the object via numerical algorithms. This allows for the extraction of both amplitude and phase information, enabling quantitative measurements of object morphology, refractive index distribution and dynamic variations [125,126]. Compared with conventional interferometric techniques, DHI can acquire three-dimensional information of a sample in a non-destructive, full-field manner; does not require chemical development; and facilitates real-time or quasi-real-time processing [127]. Reconstruction is typically based on the Fresnel–Kirchhoff diffraction integral via Fourier transform, yet recent advances include the formulation of reconstruction as an optimization problem, the application of Gerchberg–Saxton iterative algorithms, as well as the use of compressive sensing to enhance accuracy [128,129]. These methods are capable of suppressing twin-image noise, improving phase retrieval stability, and performing robustly under low signal-to-noise conditions.

The configuration of the illumination source is a key factor in enhancing DHI performance. Conventional systems employ single-wavelength coherent sources. To eliminate the 2π phase ambiguity and extend the measurable range, Min et al. [130] proposed a dual-wavelength slightly off-axis digital holographic microscopy method, which was later adapted by Di et al. [131] into a common-path configuration, thereby improving robustness and suitability for biological specimens. In recent years, a novel approach has emerged involving a single broadband source capable of simultaneously emitting numerous narrow laser lines [127], which maintains coherence while improving stability. In multi-wavelength interferometry, ΣΛ synthetic wavelength techniques [132] can reduce the equivalent wavelength to nearly half of a single wavelength, thereby enhancing measurement precision; when combined with the original ΔΛ synthetic wavelength method, a large measurement range can be preserved. However, in practice, such schemes suffer from the conjugate wavefront (twin-image) problem, which can compromise demodulation accuracy when multiplexed holograms are used [133].

In terms of optical layouts and system architecture, researchers are continuously exploring optimizations of off-axis holography, common-path arrangements, and dual-wavelength multiplexing to balance system stability and spatial resolution. For example, by encoding the sampling function with a spatial light modulator (SLM) and combining it with computational holographic reconstruction using a single-pixel detector, object information can be transmitted and recovered through complex scattering media [134].

In data processing and reconstruction algorithms, Asundi and Singh proposed one of the early methods for amplitude and phase analysis in digital dynamic holography [135]. With the advent of artificial intelligence in optical imaging, Shimobaba et al. developed a deep neural network-based approach for three-dimensional particle volume reconstruction in digital holography, significantly improving reconstruction speed and noise robustness [136]. Ren et al. further proposed an end-to-end deep learning reconstruction framework that does not require paired training data, enabling direct prediction of high-quality quantitative phase maps and substantially reducing the computation time of traditional iterative methods [137]. Furthermore, wavelet transforms, such as Cohen–Daubechies 9/7 and 17/11, have been applied for efficient compression of holographic data [138,139], considerably reducing storage and transmission demands while preserving measurement precision, thus paving the way for real-time remote holographic metrology.

Driven by these advances, DHI has expanded its applications across diverse domains. In biomedical imaging, Park et al. provided a comprehensive review of the role of DHI in label-free three-dimensional quantitative phase imaging, highlighting that holographic tomography (HT) can reconstruct the three-dimensional refractive index distribution of living cells and tissues, enabling quantitative analysis of cellular structure and functional changes [140]. Lee et al. employed three-dimensional label-free digital holographic microscopy to monitor cell migration during wound healing in real time, providing a novel tool for cell dynamics studies [141]. In fluid mechanics research, DHI combined with particle image velocimetry (PIV) and particle tracking velocimetry (PTV) enables four-dimensional reconstruction of velocity fields and particle trajectories, offering unique advantages for turbulence analysis and multiphase flow studies [142].

With its non-contact, label-free, full-field, and quantitative three-dimensional measurement capabilities, DHI has progressed from laboratory validation to engineering implementation and biomedical applications. Nevertheless, challenges remain in phase recovery accuracy under high-noise conditions, image quality for complex scattering samples, and computational demands for real-time high-resolution acquisition. Future developments are likely to focus on diversified, broadband, and coherence-controlled illumination sources; rapid, high-precision reconstruction algorithms combining deep learning with physical models; low-data-volume imaging via compressive sensing and hardware optimization; and multimodal integration (e.g., with fluorescence microscopy and photoacoustic microscopy) to obtain comprehensive datasets with structural and functional information. Breakthroughs in these directions will further expand the applicability of DHI to life sciences, materials characterization, and on-site industrial monitoring.

### 2.5. Summary

Interferometry is one of the most established optical metrology methods, relying on the phase difference generated by the interference of coherent light waves to achieve ultra-high precision displacement, surface, and refractive index measurements. Owing to its sub-nanometer accuracy and excellent sensitivity, it is widely used in fields such as semiconductor manufacturing, micro-optics fabrication, and precision surface metrology. However, interferometric techniques are highly sensitive to environmental disturbances such as vibration and temperature fluctuation, and their measurement range and robustness remain constrained. The development of phase-shifting algorithms and common-path configurations has partially mitigated these issues, but achieving both high precision and stability remains a persistent challenge.

Interferometric metrology can be effectively combined with imaging and spectroscopic methods to overcome its intrinsic limitations. For instance, integrating interferometric phase sensing with imaging modalities enables simultaneous acquisition of topographic and spatial information, while coupling with spectroscopy introduces wavelength-resolved material characterization. The convergence of interferometry with computational imaging and data-driven analysis opens new possibilities for multimodal, self-calibrating, and adaptive optical measurement systems that bridge precision metrology and intelligent perception.

## 3. Optical Imaging-Based Metrology

The technique of measuring and estimating physical properties at multiple spatial locations to form a two-dimensional or three-dimensional spatial map is referred to as imaging, which is thus distinguished from *sensing*, the latter being primarily focused on single-point measurements. Optical imaging acquires object information through the interaction between light and matter. Based on the properties of light, it can be further categorized into: (1) geometric optical imaging, including TOF, laser triangulation, and structured light; (2) computational imaging methods, including compressive sensing imaging; and (3) microscopic imaging methods.

### 3.1. Geometric Optical Imaging

#### 3.1.1. Laser Triangulation

**Figure 3:**
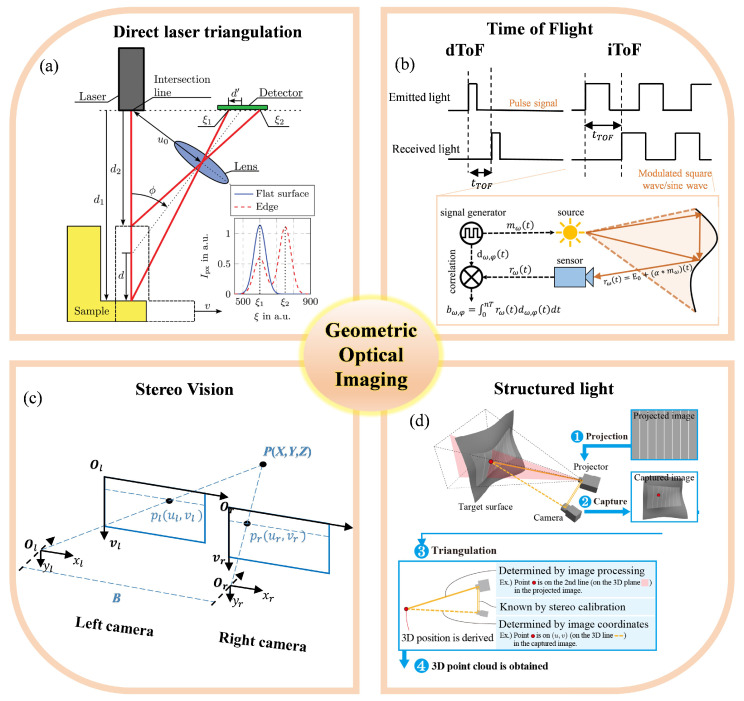
Geometric optical imaging-based metrology: (**a**) the direct type of laser triangulation; (**b**) principle of TOF distance measurment; (**c**) binocular stereo vision; (**d**) structured light.

Laser triangulation imaging is a non-contact measurement technique based on the principles of geometrical optics and triangulation. Figure 3a shows its basic principle. A laser beam is projected onto the surface of the object under inspection, and an imaging system—typically composed of a condensing lens, imaging lens, and CCD/CMOS sensor—detects the positional shift of the reflected laser spot [143], from which the spatial displacement and three-dimensional profile of the surface are calculated [144,145,146]. This approach generally employs a fixed geometric baseline, and its measurement accuracy is highly dependent on parameters such as the laser incidence angle, imaging angle, and system calibration [147]. According to the optical path configuration, laser triangulation can be classified into two types: the direct configuration, in which the optical axis is perpendicular to the measurement direction and the geometrical relationship is straightforward; and the oblique configuration, in which the relative angle between the laser source and the camera is adjusted to enhance spatial resolution and extend the measurement range, albeit with more demanding requirements for calibration and optical distortion correction [148].

The core principle relies on triangulation geometry. A laser beam illuminates the target surface, and the scattered light is imaged onto a detector (e.g., CMOS/CCD). The displacement of the laser spot on the detector correlates with the target’s vertical movement.

For a direct triangulation setup

(4) $$\Delta z=\frac{b\Delta x}{fsin\theta +\Delta xcos\theta }$$

where Δz is the target displacement, Δx is the spot displacement on the detector, *b* is the baseline distance between the laser and lens, *f* is the lens focal length, and θ is the triangulation angle.

In recent years, significant progress has been made in error modeling and compensation strategies to address challenges in the measurement of complex surfaces, such as diverse reflective properties, color variations, and surface inclination. Ding et al. [144] proposed a two-stage compensation approach based on the Lambert illumination model, achieving measurement error reductions of 71.5% and 91.9% in the coarse and fine stages, respectively. Li et al. [149] established an optical error compensation model using least squares and functional libraries, reducing the measurement error by approximately 17%, although the method remained limited for surfaces deviating from ideal diffuse reflection. Yu et al. [150] employed the Phong reflection model to account for both specular and diffuse reflection components, enabling more precise compensation for complex reflective surfaces. Hao et al. [147] investigated the influence of rough-surface scattering on displacement measurement accuracy in position-sensitive detector (PSD)-based triangulation systems and proposed optimization strategies for the detection principle. In addition, studies have shown that dual-view triangulation can partially suppress speckle noise during scanning operations, thereby improving measurement robustness [145].

With advances in computational technology, conventional geometric modeling and optical compensation approaches are increasingly combined with intelligent algorithms. For example, convolutional neural networks (CNN) and deep learning-based methods have been introduced for feature extraction and signal fitting [151], allowing for robust performance under varying material properties and surface morphologies with associated intensity fluctuations and noise. Moreover, the application of this technique in on-machine measurement (OMM) has expanded substantially. By integrating OMM with multi-axis machine tool platforms and optimizing measurement path planning [152,153,154], not only has measurement efficiency been enhanced, but coverage and accuracy for large-scale freeform surfaces and complex structural components have also been improved.

Benefiting from advantages such as non-contact operation, high resolution, and rapid response, laser triangulation imaging has found widespread applications in precision manufacturing, optical inspection, and large-scale structural metrology. Nevertheless, its performance remains constrained by the stability of optical components, environmental influences (e.g., vibration and temperature variation), and the optical properties of the measured surfaces [147,149]. Future developments are likely to focus on multi-modal hybrid metrology (combining structured light, interferometry, and other modalities), intelligent error compensation (leveraging AI to optimize modeling and data processing), and customized system architectures for specific application scenarios, with the aim of achieving higher accuracy, broader adaptability, and superior capability for real-time, in-process measurement.

Time-of-Flight (ToF) imaging is an active three-dimensional (3D) sensing technology that estimates the distance to an object by emitting a light signal with known modulation characteristics and measuring the propagation time of its reflection from the target. This technique does not rely on surface texture features, can operate under low-light or even no-light conditions, and is capable of capturing full-scene depth information within a single frame.

Depending on the measurement principle, ToF imaging can be categorized into direct time-of-flight (dToF) and indirect time-of-flight (iToF) methods. Figure 3b shows the principle difference between iToF and dToF. The dToF approach employs pulsed light and precisely measures the time delay between emission and reception, whereas iToF uses continuously modulated light and derives distance from the measured phase shift. These two techniques differ significantly in light source design, detector architecture, signal processing methods, and performance metrics. However, with recent advancements in semiconductor devices, light source modulation strategies, and computational imaging algorithms, their application scenarios and performance boundaries have been continuously expanded, and a trend toward technological convergence has emerged.

dToF technology determines the distance by directly measuring the round-trip time delay of a light pulse, expressed as

(5) $$d=\frac{c\cdot \Delta t}{2}$$

where *c* denotes the speed of light and Δt is the round-trip propagation delay of the light signal. A typical hardware architecture includes a pulsed laser source, optical transmitter, optical receiver, high-sensitivity photodetector, and a high-resolution time-to-digital converter (TDC). In recent years, single-photon avalanche diodes (SPADs) have been widely adopted in the receiver front end of dToF systems. When combined with picosecond-pulsed lasers, SPADs significantly improve detection sensitivity and timing resolution, enabling millimeter-level or even micrometer-level ranging precision [155,156].

In high-performance dToF research, a 256 × 192-pixel CMOS receiver using a current-integrating transimpedance amplifier (CI-TIA) as an analog front-end can suppress the direct current component caused by strong background light during pulse detection, achieving < cm constant error and <0.09% precision over a 240 m ranging distance, while maintaining 30 frames/s imaging capability and offering strong performance in high dynamic range and long-distance scenarios [157]. For medium-range, high-frame-rate applications, combining a two-dimensional mechanical scanning architecture with pseudo-random coding achieves 16 frames/s at 4.5 m range, with 0.2% precision, and suppresses interference levels by more than 15.29 dB [158]. To further reduce power consumption and system complexity, hybrid architectures have been proposed that integrate partial histogram accumulation, adaptive pulse-width adjustment, and nonlinear spatiotemporal coincidence detection (NSCD), enabling sustained 60 m measurement under 60 klux background light with sub-centimeter precision, while compressing per-pixel histogram storage to just 180 bits [159].

Exploration of ultimate dToF precision has demonstrated that, by tuning SPAD bias voltage, laser repetition rate, and integration time, micrometer-level ranging precision can be achieved [156]. Overall, dToF offers high accuracy, excellent sensitivity, and long-range measurement capability, making it particularly suitable for LiDAR, precision mapping, and low-illumination 3D imaging. However, the use of high-speed TDCs, high-peak-power pulsed light sources, and high-speed readout chains contributes to higher costs and power consumption, posing challenges for large-scale miniaturization in consumer electronics.

#### 3.1.2. Time-of-Flight Imaging

iToF technology employs a continuous-wave modulated light source, where distance is derived from the measured phase shift ϕ between the emitted and received optical signals:

(6) $$d=\frac{c}{4\pi f_{m}}\phi$$

Here, $f_{m}$ is the modulation frequency. Based on lock-in detection principles, iToF pixels can be fully integrated in CMOS processes with per-pixel phase measurement capability, thereby capturing two-dimensional phase information with relatively low cost and power. This makes iToF popular for medium- to short-range applications such as consumer electronics, robotic vision, and indoor mapping [155].

Recent studies have advanced iToF accuracy and robustness. In hardware, the proposed eight-window continuous-wave iToF (cw-iToF) method replaces the conventional sinusoidal modulation with a square-wave source and extends the number of integration windows from four to eight. This approach reduces phase nonlinearity error significantly, resulting in a worst-case error of 1.2 mm over 0.75 m range, with more than 90% of the measurement range keeping errors below 1 mm, and achieving 0.35 mm precision with a 100 ms frame time [160]. A parallel-phase-demodulated AMCW scanning sensor can dramatically shorten total integration time while maintaining high demodulation contrast. Using only 30 mW of optical power, it achieves a relative noise of 0.056% at a 1.5 m reference distance, and enables 1920 × 1080 full-HD 3D depth imaging within just 800 ns of integration [161].

To address multipath interference (MPI), a key weakness of iToF, researchers have proposed synthetic dataset generation using an accurate light propagation model, combined with machine learning. A Bayesian-optimized XGBoost model trained on such data reduces MPI mean absolute error (MAE) in coaxial-scanning AMCW LiDAR to the millimeter scale, maintaining an MAE of just 2.8 mm in sharp-corner object scenes [162]. Furthermore, data-driven post-processing for error compensation has been shown to significantly improve ranging accuracy under indoor conditions, outperforming traditional correction approaches without adding optical or hardware complexity [163].

In summary, iToF systems offer excellent hardware integrability, low cost, and high-frame-rate imaging, well suited for high-resolution, medium- to short-range 3D sensing. However, their performance is inherently limited by modulation frequency, phase ambiguity, and MPI sensitivity, making them less stable than dToF in long-range or high-interference scenarios. Nevertheless, recent advances in multi-window demodulation, parallel processing, high-frequency square-wave modulation, and AI-based error compensation point towards promising routes for bridging the performance gap with dToF.

#### 3.1.3. Stereo Vision

Binocular vision is a passive three-dimensional perception technique based on bionic principles. It simulates human binocular disparity using a pair of cameras and applies triangulation to compute depth information. The core of this method lies in reconstructing the three-dimensional structure of the scene by disparity matching between left and right images, without the need for projecting an active light source. As such, it is well-suited for dynamic scenes and complex environments.

In a parallel stereo camera configuration, intrinsic parameters (focal length, distortion coefficients) and extrinsic parameters (rotation matrix *R*, translation vector *T*) are obtained through calibration. Feature point matching (e.g., SIFT/SURF) is then performed to generate a disparity map, which is finally used for 3D reconstruction. The principle follows:

(7) $$Z=\frac{BF}{d}$$

where *Z* is the target depth, *B* is the baseline (distance between the optical centers of the two cameras), *F* is the focal length, and *d* is the disparity between corresponding points in the left and right images. Pixel similarity is typically computed using SAD, SSD, or Census Transform, with disparity refinement achieved via guided filtering, dynamic programming, and other approaches. Disparity is then computed by applying Winner-Takes-All (WTA) or global optimization algorithms.

A stereo vision system consists of two cameras and a synchronized triggering device. In industrial scenarios, sub-millimeter accuracy (±0.1 mm) can be achieved; for example, PCB pin-tip 3D reconstruction can reach an accuracy of ±0.05 mm. In virtual reality (VR), conventional binocular vision enables the generation of high-accuracy 3D environment models.

Conventional stereo vision is a mature technology, with standardized algorithms (e.g., BM, SGM) supporting large-scale applications. However, its accuracy depends heavily on calibration, and environmental temperature variations can induce baseline drift, requiring frequent recalibration. Moreover, in low-texture regions—such as walls or specular surfaces—matching errors are likely to occur.

To address the limitations of conventional stereo vision, several variants have emerged, such as mirror-based stereo vision [164] and stereo zoom super-resolution. Mirror-based stereo vision uses a single camera combined with a reflective mirror assembly (e.g., curved mirrors, prisms) to simulate two viewpoints. Through optical design, system volume can be reduced by more than 50%, and the configuration offers resistance to vibration and tolerance to extreme temperatures, making it suitable for confined spaces in UAVs, in-vehicle systems, and dynamic industrial robotic scenarios. However, distortion correction is challenging due to non-linear image distortion caused by mirror surface shape errors, which necessitate complex calibration algorithms. In addition, the optical path alignment is highly sensitive, as assembly deviations in the mirror system directly affect measurement accuracy.

Stereo zoom super-resolution systems dynamically adjust the focal length of cameras and combine this with super-resolution algorithms (e.g., SRCNN, ESRGAN) to enhance the resolution of distant details. Advantages include high-precision long-range measurement, support for wide-angle environmental perception, and local detail magnification, making it suitable for traffic analysis [165]. Disadvantages include high computational complexity—super-resolution algorithms may require over 100 TOPS of processing power, limiting real-time performance—and high hardware cost, as precision zoom lenses are typically 3–5 times more expensive than fixed-focus lenses.

At present, AI-enhanced techniques represent a major development trend in binocular vision. End-to-end stereo matching networks based on convolutional neural networks (CNNs), such as GCNet and PSMNet, have significantly improved matching accuracy in low-texture regions. In parallel, lightweight design and real-time optimization are also key directions. Researchers aim to package stereo systems into portable modules, combining optical flow with stereo matching to address motion blur, thereby extending applications to industrial inspection and medical navigation.

#### 3.1.4. Structured Light 3D Reconstruction

Structured-light-based three-dimensional (3D) reconstruction is an important active optical measurement technique [166,167]. By projecting specifically designed structured gratings or fringe patterns onto the surface of the measured object, and acquiring their deformed images via a camera, the object’s 3D shape can be computationally recovered [168,169,170,171]. Compared with passive stereo vision methods, which rely on natural textures and feature extraction, structured light maintains high robustness and measurement accuracy even under conditions of low texture, poor illumination, and occlusion [172,173]. Consequently, it has been widely applied in industrial surface inspection, facial recognition, and 3D modeling, among other fields [174,175,176,177].

Among the various implementation approaches, fringe-based structured light 3D reconstruction has become the dominant technique owing to its high resolution, high accuracy, and flexible system configuration. Typical methods include Fringe Projection Profilometry (FPP) and Phase Measuring Deflectometry (PMD).

FPP is a phase-encoding-based active optical 3D measurement method, suitable for high-precision measurement of diffuse reflective surfaces [178,179,180]. In FPP, periodic sinusoidal fringe patterns are projected onto the object’s surface under known geometric conditions. The fringes are distorted by the surface geometry, and the deformed patterns are captured by a camera. By decoding the fringe phase, the 3D geometry of the object can be reconstructed [181]. To establish the mapping between the extracted phase and the real-world 3D coordinates, two common calibration models are employed: the phase–height model and the triangulation model. In the phase–height model, multiple reference planes with known heights are used to directly establish a functional relationship between phase and height, making it suitable for relatively flat surface measurements. Common mathematical forms include linear, inverse-linear, and polynomial models [182,183,184,185]. The triangulation model, on the other hand, requires precise calibration of the camera and projector to recover the object’s 3D coordinates using the principles of triangulation. Depending on projection mechanisms, FPP systems can be implemented in various ways. The most widely used approach relies on Digital Light Processing (DLP) projectors, which offer high pattern quality and fast refresh rates, making them a primary choice. However, as DLP projection relies on focusing optics, it inherently suffers from a limited depth of field. To address this limitation, more compact Micro-Electro-Mechanical Systems (MEMS)-based systems have been developed, which scan laser beams to form patterns, allowing large-depth-of-field projection without the need for focusing optics [167,186].

PMD is a technique specifically designed for the 3D reconstruction of specular or highly reflective surfaces. A typical PMD system consists of a liquid crystal display (LCD), a camera, and a computer. The computer generates sinusoidal fringe patterns on the LCD, which are reflected by the object’s specular surface and then captured by the camera. Due to the geometric modulation caused by surface normals, the recorded images encode phase distortions that correspond to local surface slope variations [187,188]. In the reconstruction process, wrapped phases are first extracted from the captured fringe patterns, typically using phase-shifting methods. These phases are then converted into surface gradient data through the use of geometric models and calibration parameters [189]. Since the phase distortion is proportional to the reflected ray deflection angle, PMD yields a gradient field representing the surface slope. To recover a complete 3D surface, the gradient field must be numerically integrated over the 2D image plane, producing the relative height map [190]. To simplify this process, Direct Phase Measuring Deflectometry (DPMD) has been proposed, which employs a dual-LCD and dual-camera setup to simultaneously capture fringes reflected from a reference plane and from the measured specular surface. By comparing the phase difference along the two reflection paths, height variations can be directly derived, thus avoiding the complex numerical integration steps required in conventional PMD, and enabling direct height reconstruction for specular targets [191,192].

Despite differences in system architectures, both FPP and PMD operate by projecting or displaying sinusoidal fringe patterns and exploiting the modulation effects imposed by the measured object to recover 3D shape information [193,194]. Once the fringes are distorted by the object’s surface, the phase variations in the patterns directly encode spatial geometric features. In FPP, the extracted phase is directly related to the object’s depth, whereas in PMD, it corresponds to the object’s surface gradients. High-precision phase extraction and phase unwrapping are therefore essential for both techniques. Common wrapped phase retrieval methods include phase-shifting [195], wavelet transform [196], and Fourier transform methods [197]. Based on the dimensional domain of information used in the unwrapping process, phase unwrapping approaches in structured light 3D reconstruction can be broadly categorized into temporal phase unwrapping (TPU) and spatial phase unwrapping (SPU). TPU relies on projecting multiple fringe patterns with different frequencies or coding schemes, and computes the absolute phase for each pixel independently from intensity variations over time. Since TPU does not rely on spatial continuity, it exhibits high robustness when dealing with surface discontinuities, abrupt depth changes, or occlusions [198]. In contrast, SPU depends on the phase relationships between neighboring pixels, progressively removing the 2π discontinuities to recover the true phase. SPU methods can be classified as either local or global approaches [199]. However, noise in SPU can propagate from high-noise areas to low-noise ones, potentially degrading accuracy.

Traditional fringe-projection structured light systems often suffer from reduced measurement accuracy when dealing with objects having non-uniform surface reflectivity, highly complex geometry [200], or severe occlusion [201]. In recent years, deep learning—demonstrated to be powerful in feature extraction and nonlinear modeling—has been successfully applied in numerous domains [65,202,203,204]. For fringe-projection-based structured light systems, deep learning offers promising solutions to improve measurement accuracy, accelerate reconstruction speed, and enhance robustness [65,202,205,206]. Nevertheless, structured light 3D reconstruction continues to face multiple challenges in practical applications, including: maintaining high accuracy and stability in complex and dynamic environments [207,208]; mitigating measurement errors caused by overexposure or underexposure under high dynamic range conditions [209]; achieving real-time reconstruction under limited depth of field and fast motion; and enhancing computational efficiency without sacrificing accuracy to meet the demands of industrial online inspection and intelligent manufacturing.

### 3.2. Computational Optical Imaging

Computational Imaging (CI) is an imaging paradigm that integrates optical design with computational post-processing. By jointly optimizing optical hardware and algorithms, CI overcomes the physical limitations of conventional imaging systems. Its core idea is to reconstruct high-dimensional, high-resolution, or attribute-specific images from indirect or incomplete measurements through physical modeling and mathematical optimization [194,210]. CI methods can mitigate optical noise, diffraction limits, or dynamic range constraints, while also simplifying hardware complexity via computational compensation, and further enabling tasks unattainable by traditional imaging (e.g., imaging through scattering media, light-field reconstruction).

Its mathematical model can be expressed as:

(8) y=Φx+n

where Φ denotes the measurement matrix, and *n* represents noise.

**Figure 4:**
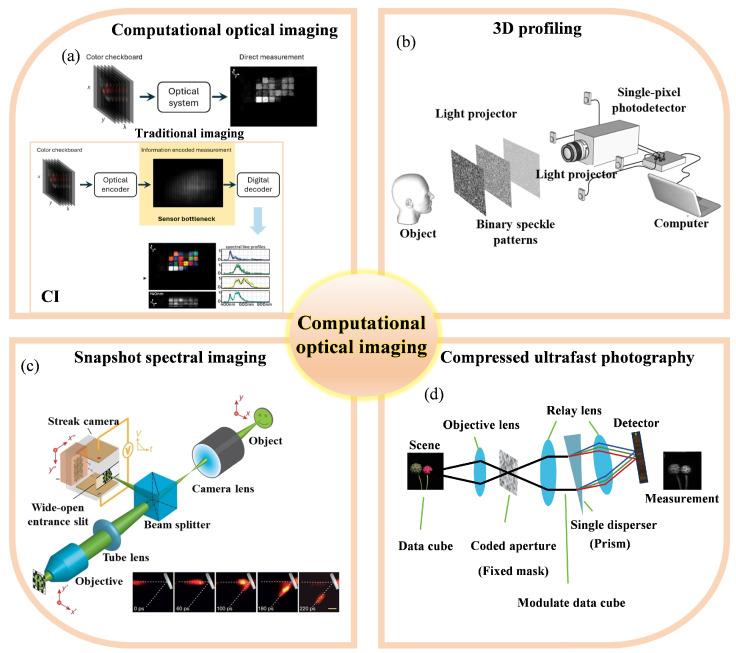
Representative applications of computational imaging (CI): (**a**) comparison of conventional and computational optical imaging architecture; (**b**) CI systems employing multiple single-photon detectors for 3D profiling; (**c**) single-disperser coded aperture snapshot spectral imaging (CASSI); (**d**) compressed ultrafast photography (CUP).

Computational imaging encompasses a wide range of techniques. Existing surveys categorize them according to encoding/modulation strategies, dimensional expansion, or application-driven classification. As illustrated in Figure 4, there are numerous types of CI applications. This work focuses on industrial and entertainment fields, highlighting three representative subcategories.

#### 3.2.1. Compressed Imaging

Compressed imaging is based on the theory of Compressed Sensing (CS), whose central premise is to exploit the sparsity of a signal in a certain transform domain to achieve image acquisition and reconstruction at sampling rates significantly below the Nyquist rate. Let the original image $x\in R^{N}$ be represented under a sparsifying basis Ψ as x=Ψs, where the coefficient vector s is sparse or approximately sparse. Compressed measurements are obtained via a measurement matrix $\Phi \in R^{M\times N}$ (M≪N):

(9) y=Φx=ΦΨs

Image reconstruction is typically accomplished by solving a constrained optimization problem:

(10) $$\min_{s}{∥s∥}_{1}\;\;\mathrm{subject}\;\mathrm{to}\;\;∥y-\Phi \Psi s{∥}_{2}\leq \epsilon ,$$

where ϵ denotes the noise tolerance. This framework overcomes the limitations imposed by traditional imaging systems on sampling rates and detector requirements.

A typical compressive imaging system is the single-pixel camera (SPC), which employs a digital micromirror device (DMD) to spatially encode the incident optical field [211]. For each measurement, a single photodetector records the corresponding linear combination of the encoded light intensity, thereby producing the compressed sampling result. This architecture is particularly advantageous in scenarios where the detected signal intensity is severely attenuated due to scattering or absorption, such as in biomedical imaging [212,213] or long-range three-dimensional imaging [214,215].

In recent years, extensive research has been conducted on both algorithms and applications of compressive imaging. On the algorithmic side, beyond traditional convex optimization and greedy algorithms [216,217], deep learning-based approaches have become increasingly dominant. Reconstruction methods leveraging deep neural networks, such as the deep residual reconstruction network (DR2-Net) and the bidirectional recurrent neural network architecture (BIRNAT), have demonstrated significant improvements in both reconstruction speed and accuracy compared to conventional approaches [218,219].

On the application side, compressive imaging has found broad adoption in hyperspectral imaging [213,220], holography [221], polarimetry [222], and multi-modality imaging [223]. For instance, in hyperspectral imaging, Hahn et al. proposed a method that integrates compressive sensing (CS) with adaptive direct sampling (ADS), enabling the acquisition of high-quality hyperspectral images with substantially fewer samples than the number of pixels. Their experiments on real datasets showed that even with only ∼40% of the samples, excellent image quality and classification accuracy could still be achieved [220]. In the field of holography, Clemente et al. introduced the concept of single-pixel imaging into digital holography by combining phase-shifting interferometry with a single-pixel detector. Using Hadamard patterns to compressively encode the object’s diffraction field within a Mach–Zehnder interferometer, they employed back-propagation algorithms to reconstruct the complex amplitude of the object and successfully demonstrated phase distribution measurements of ophthalmic lenses [221].

Benefiting from its advantages in hardware simplification, reduced sampling requirements, and multi-dimensional imaging capability, compressive imaging has emerged as a key research direction in modern optical metrology. Future developments are expected to focus on the design of efficient and low-complexity reconstruction algorithms, the integration of deep learning with physics-based models, and the expansion of compressive imaging toward high-dimensional and multi-modal optical measurement tasks.

#### 3.2.2. Light Field Imaging

A light field provides a comprehensive description of the propagation state of light in space, encompassing not only intensity but also directional information. When represented using the Two-Plane Parameterization (2PP) model, a light field can be described by a four-dimensional function L(u,v,s,t), where (u,v) denote the coordinates on the camera imaging plane, and (s,t) correspond to the intersection points on the object plane, enabling a full capture of both spatial position and propagation direction. This property confers inherent advantages for acquiring three-dimensional structural information, optical wavefront data, and post-capture refocusing with extended depth-of-field.

Microlens array light field cameras introduce a microlens array between the main lens and the image sensor, allowing angular resolution of incident light rays [224,225]. This approach offers a compact structure and full light field capture, making it widely applicable in digital photography and microscale 3D imaging [226,227]. However, the trade-off between spatial and angular resolution, constrained by microlens size and sensor pixel density, remains a primary limitation affecting measurement accuracy.

Camera arrays capture images from multiple spatially distributed viewpoints to reconstruct dense light fields [228,229]. This method can provide high spatial and angular resolution, suitable for large-scale 3D scene reconstruction and dynamic scene capture [230]. Nevertheless, camera arrays face challenges in hardware cost, system calibration, and data synchronization, limiting their adoption in precision metrology.

Coded aperture and computational light field imaging employ spatial light modulators (SLMs), phase masks, or specially designed coded optical elements to embed angular information into captured images, followed by computational inversion to reconstruct the light field [231,232]. Compared with conventional approaches, coded light field imaging can achieve relatively high spatial and angular resolution without significantly increasing hardware complexity and provides flexible imaging modes [233]. Recently, deep learning-based reconstruction methods have further enhanced both reconstruction accuracy and real-time performance of coded light field systems [234].

In optical metrology, light field imaging enables the acquisition of viewpoint variation and depth cues inaccessible to conventional imaging. Combined with algorithms such as refocusing, parallax estimation, and epipolar plane image (EPI) feature extraction, it can achieve high-precision three-dimensional surface reconstruction and profilometry [235,236,237].

Since its introduction by Ng et al. in 2005, light field imaging has been gradually applied in computational photography [224,238]. Ihrke et al. provided a comprehensive review of 25 years of light field research, highlighting the importance of joint optimization of optical design and computational reconstruction [239]. Hu et al. systematically reviewed the development of light field cameras for metrology, discussing imaging principles, calibration, reconstruction algorithms, and applications in 3D measurement and micro/nanoscale inspection [240]. In fluid mechanics, Shi et al. summarized applications of light field cameras for volumetric flow measurements and turbulent combustion diagnostics, emphasizing their compact structure and capability to acquire full 3D information in a single exposure, thereby addressing limitations of conventional stereo vision and multi-camera array systems [241]. Broxton et al. extended light field microscopy to micrometer-scale 3D imaging of biological specimens [226], while Prevedel et al. combined light field capture with computational reconstruction to record rapid neuronal activity in three dimensions [227].

Despite its advantages in non-contact 3D measurement, freeform surface inspection, and reconstruction of transparent and reflective materials, light field imaging still faces challenges. Spatial and angular resolution limitations constrain high-precision applications, while the large volume of light field data demands high computational efficiency and real-time processing. To address these issues, several strategies have been proposed:

High-resolution and super-resolution reconstruction: To mitigate resolution loss in microlens array cameras caused by effective pixel sparsity, sparse reconstruction and deep learning-based super-resolution techniques have been developed. For example, combining convolutional neural networks (CNNs) with sparse coding allows enhanced spatial sampling while preserving angular information, enabling sub-pixel 3D reconstruction [234,242].

Computational light field and deep learning inversion: Advances in computational optics and deep learning enable embedding light field information in hardware via coded apertures or phase masks, followed by rapid, high-precision reconstruction and depth estimation using deep neural networks, such as EPI convolution networks or NeRF-based implicit representations [233,243].

Dynamic light field capture and online metrology: For real-time 3D inspection of dynamic objects in industrial production, high-speed light field capture systems using multi-view arrays and temporal encoding have been developed, leveraging GPU parallelization and edge computing to achieve high-frequency 3D measurement and defect detection [244,245].

Light field and wavefront sensing integration: Chen et al. proposed a wavefront measurement method based on an optimized light field camera, achieving high-precision wavefront acquisition in a single exposure without complex interferometric modulation, suitable for freeform optical element characterization [246].

Cross-scale and multi-modal fusion metrology: Integration of light field imaging with structured illumination, confocal microscopy, and interferometry enables high-precision 3D measurements across micro-to-nanoscale, improving robustness for complex surfaces and transparent or reflective materials [247,248,249].

In summary, while light field imaging offers significant advantages in optical metrology, challenges remain, including the trade-off between spatial and angular resolution, large data volume and processing demands, and hardware scalability. Future developments in super-resolution reconstruction, intelligent hardware, and computational optics, combined with AI-augmented physics-based reconstruction methods, are expected to expand its applications in micro/nano manufacturing, precision metrology, and biomedical imaging, enhancing both measurement accuracy and system performance.

### 3.3. Super-Resolution Imaging

In conventional optical microscopy, image resolution is fundamentally constrained by the diffraction limit. When a point source is imaged through an ideal lens, it produces a finite-sized intensity distribution on the image plane, known as the point spread function (PSF). The lateral resolution of a microscope is typically characterized by the full width at half maximum (FWHM) of the PSF, which can be approximated as FWHM≈0.61λ/NA, where λ denotes the wavelength of light and NA represents the numerical aperture of the objective lens. Within the visible spectrum, the use of high-NA oil immersion objectives (e.g., NA = 1.40) yields a conventional optical resolution of approximately 200 nm. However, in fields such as nanostructure characterization and biological imaging, the demand for higher resolution has become increasingly pressing. To overcome the diffraction limit, a variety of super-resolution (SR) imaging techniques have been developed, enabling enhanced lateral and axial resolution beyond that of traditional optical systems [250,251,252].

#### 3.3.1. Near-Field Super-Resolution Imaging

Near-field scanning optical microscopy (NSOM) represents one of the earliest optical techniques to overcome the diffraction limit. Its fundamental principle lies in probing the evanescent field in the immediate vicinity of the sample surface, thereby retrieving high-spatial-frequency information. Conventional optical systems are unable to capture such information; however, by positioning a miniature probe (e.g., a tapered optical fiber or metallic tip) within the evanescent field, the non-radiative light can be converted into detectable radiation via scattering or coupling, enabling nanometer-scale imaging [253,254].

The most common implementations of NSOM include the use of aperture probes based on tapered optical fibers [255], vibrating metallic tips [256], and apertureless approaches such as photon scanning tunneling microscopy (PSTM) [257]. These approaches employ different near-field detection strategies to achieve spatial resolutions far beyond those of conventional optical systems. In particular, aperture-type SNOM systems have achieved lateral resolutions on the order of tens of nanometers or better. Heinzelmann et al. demonstrated detection at incidence angles larger than the critical angle for total internal reflection, thus overcoming conventional optical limits and achieving improved imaging performance [258]. Hecht and co-workers subsequently provided a systematic overview of aperture-based SNOM imaging principles, outlining multiple system configurations and optimization strategies [255]. To further enhance resolution and extend applicability, researchers have developed apertureless probe techniques, including metallic tips exploiting localized field enhancement [259], tetrahedral probes [260], and non-contact scanning systems integrated with interferometric detection. These apertureless SNOM platforms can achieve spatial resolutions down to 10 Å, representing the state of the art in optical microscopy.

In the field of manufacturing metrology, NSOM has been successfully applied to the characterization of sub-20 nm semiconductor structures and to measurements of residual layer thickness in nanoimprint lithography. For instance, Takahashi et al. demonstrated the use of metallic tips exploiting near-field enhancement for precise detection of residual layers at the 10 nm scale [261]. Ohtsu et al. reported a photon scanning tunneling system employing a nanofiber probe, which achieved axial resolutions of only a few nanometers [262].

With advances in probe fabrication, probe–sample distance control, and vibration isolation systems, commercial instruments are now capable of achieving resolutions on the order of 10 nm. Notably, the neaSNOM system developed by Neaspec integrates multiple near-field detection modes and has been widely employed in materials science and semiconductor research [263]. Despite its remarkable spatial resolution, NSOM remains limited by several inherent challenges: (1) the imaging area is constrained by the short interaction range of the near field (typically only tens of nanometers); (2) the imaging speed is restricted by the point-by-point scanning mechanism; and (3) system stability and tip degradation remain persistent engineering concerns. Consequently, NSOM is currently regarded more as a high-precision complementary technique for micro- and nanostructure characterization rather than a replacement for large-area optical metrology tools.

#### 3.3.2. Pupil-Filtering Confocal Super-Resolution Imaging

Aperture-filtering imaging technology originates from the engineered design of the point spread function (PSF) in Fourier optics. It was first proposed by Di Francia in 1952, who pointed out that by controlling the propagation characteristics of incident light in the frequency domain, it is possible to overcome the diffraction limit of conventional optical systems and thereby enhance resolution [264]. Typically, this approach employs specially designed diaphragms or phase plates—such as annular apertures or segmented masks—placed in the Fourier plane of the optical system to modulate the amplitude or phase of the light field. Such modulation compresses the main lobe and suppresses side lobes of the PSF, resulting in improved lateral resolution [265,266].

In recent years, aperture-filtering techniques have been increasingly integrated with confocal microscopy. Owing to its superior optical sectioning capability and effective background suppression, the confocal microscope provides an excellent platform for super-resolution imaging. When combined with aperture-filtering structures, it can substantially sharpen image features and further enhance resolution. For example, Zhao et al. proposed a bipolar annular aperture coupled with an absolute-differential confocal detection scheme, which effectively narrowed the PSF main lobe and achieved resolution improvements beyond 0.2 μm [267]. The critical advantage of this approach lies in its simultaneous suppression of side lobes and enhancement of axial contrast, making it highly suitable for precise measurements of fine structures. Similarly, Tang et al. demonstrated that radially polarized illumination combined with a multi-zone phase plate can produce a tighter focal spot and extended depth of focus [266]. This unique combination of tight focusing and long focal depth makes the technique advantageous for applications in three-dimensional structural inspection and deep-tissue imaging. Comparable strategies have also been applied in high-precision domains such as MEMS displacement metrology and curved-surface profilometry [268,269].

On the instrumentation side, Arrasmith et al. developed a MEMS-based handheld confocal microscope incorporating an aperture-modulation module, which enabled in vivo skin imaging and demonstrated the potential of aperture-filtered confocal microscopy for portable medical devices [270].

At the theoretical level, Sun and Liu proposed that phase modulation combined with polarization control can be used to design optical fields that generate ultrafine focal spots while maintaining extended depth of focus, thereby providing theoretical guidance for subsequent aperture-filter design [271].

Overall, aperture-filtered confocal imaging provides an effective route to overcoming the resolution limitations of conventional confocal microscopy by manipulating light propagation in the spatial frequency domain. Nevertheless, several challenges remain, including the inherent trade-off between main-lobe compression and side-lobe enhancement, the increased complexity of the optical setup, and significant light throughput loss, which can ultimately degrade the signal-to-noise ratio in imaging.

#### 3.3.3. Structured Illumination Microscopy

Structured illumination microscopy (SIM) is among the most widely applied super-resolution imaging techniques, particularly demonstrating outstanding performance in live-cell imaging and micro/nanofabrication inspection. The fundamental principle of SIM lies in projecting a predefined spatially modulated illumination pattern (e.g., sinusoidal fringes, gratings, or lattices) onto the specimen. This process induces Moiré interference, which mixes high-frequency sample information—originally beyond the optical system’s passband—into the lower-frequency domain, thereby enabling its retrieval during subsequent image reconstruction [272,273].

Compared with point-scanning-based super-resolution methods such as Stimulated Emission Depletion Microscopy (STED) and Photoactivated Localization Microscopy (PALM)/Stochastic Optical Reconstruction Microscopy (STORM), Structured Illumination Microscopy (SIM) offers advantages including faster imaging speed, reduced phototoxicity, and suitability for thick specimens and live-cell observations. Moreover, since the excitation intensity required for SIM is significantly lower than that of STED, it is particularly advantageous for long-term imaging and the recording of dynamic processes. In the field of materials science and micro/nanomanufacturing, SIM has also been employed for high-precision detection of nanoscale targets such as surface structures, subwavelength defects, and lithography residues. For instance, Takahashi et al. utilized infrared standing-wave structured illumination to achieve 100 nm resolution in the detection of nanoscale defects on silicon wafer surfaces [274].

To further enhance resolution, researchers have developed advanced approaches such as saturated structured illumination microscopy (SSIM) and nonlinear SIM. These methods exploit the saturation response or nonlinear modulation of fluorophores to extend the system’s frequency support beyond the range accessible to conventional SIM, theoretically pushing the resolution below 50 nm. For example, the SSIM model proposed by Gustafsson has been experimentally demonstrated to surpass the spatial resolution of linear SIM by more than twofold [275]. More recently, deep learning and data-driven strategies have been integrated into SIM systems to reduce the number of raw frames required for reconstruction, improve imaging quality under low signal-to-noise conditions, and accelerate acquisition speed. Such approaches not only simplify experimental implementation but also enhance system robustness against noise and misalignment [273,276].

Despite its success, the practical deployment of SIM in engineering applications still faces several challenges. The illumination patterns typically rely on interference fringes or spatial light modulators (SLMs), requiring high optical stability and precise system calibration. In addition, the reconstruction process involves Fourier-domain processing and multi-frame fusion, which are not inherently fault-tolerant. Furthermore, high-resolution reconstructions often require multiple phase-shifted and angularly rotated images, resulting in relatively long acquisition times that limit the applicability to high-throughput inspection. Consequently, current research has focused on developing novel SIM variants that enable high speed, reduced frame acquisition, and stronger resilience to disturbances. Approaches such as adaptive fringe illumination, compressed-sensing-based reconstruction, and AI-assisted image recovery are being actively explored to meet the demands of industrial inspection and real-time monitoring [276].

#### 3.3.4. Micro-Object-Based SR Imaging

Since the concept of the Photonic Nanojet (PNJ) was first introduced by Chen et al. in 2004, this technique has rapidly advanced in the field of super-resolution imaging [277]. A PNJ refers to a highly localized, high-intensity, non-diffracting light beam formed on the shadow side of a micron-scale transparent dielectric microparticle (e.g., a glass microsphere) when illuminated by an incident light. Unlike conventional near-field optical methods, PNJ-based imaging does not rely on probes or nanoscopic apertures; instead, it exploits the capability of microstructures to manipulate the optical field, enabling high-resolution focusing directly in the far-field region. This approach combines ease of system implementation with high imaging precision. The focusing performance of PNJs is influenced by parameters such as the refractive index and size of the microsphere, as well as the surrounding substrate medium, all of which can be optimized through engineering [278,279].

Simulations based on Mie scattering theory and angular spectrum propagation theory indicate that PNJs can achieve focal spots with full width at half maximum (FWHM) below 0.5λ, while maintaining high focal intensity and strong energy localization [278,279]. Ferrand et al. experimentally visualized the spatial distribution and focusing characteristics of PNJs, confirming their practical potential [280]. Wang et al. developed a white-light nanoscopy system using 3 μm-diameter silica microspheres, achieving approximately 50 nm lateral resolution under far-field conditions, representing a notable demonstration of PNJ-based imaging in practice [281]. Furthermore, researchers have extended the approach at the material and structural level; for example, Lee et al. proposed using self-assembled microsphere arrays to realize near-field focusing and magnification, further enhancing imaging resolution and field of view [282].

In practical applications, PNJ technology has been widely employed for biological sample imaging, subsurface defect inspection, nanolithography, and surface-enhanced Raman spectroscopy (SERS) [252]. Its far-field nature facilitates integration into existing optical systems, offering high portability and commercial potential. Compared with conventional super-resolution methods, microstructure-based imaging provides advantages such as minimal requirement for complex nanofabrication, low cost, and flexible system assembly, making it particularly suitable for integration with standard microscopy platforms [252]. Nevertheless, this approach also has certain limitations. The imaging performance is highly sensitive to the contact state between the microsphere and the sample, favoring predominantly two-dimensional specimens, and precise positioning and manipulation of the microspheres still rely on mechanical methods, limiting high-precision automation.

### 3.4. Summary

Imaging-based optical metrology techniques rely on the analysis of intensity or phase information in captured images to reconstruct geometric and spatial characteristics of objects. Typical methods include confocal microscopy, digital holography, and structured-light 3D measurement. These techniques offer high flexibility, wide field of view, and non-contact measurement capabilities, making them well suited for surface profiling, biological imaging, and industrial inspection. Compared to interferometric approaches, imaging-based methods are more robust against environmental perturbations but generally exhibit lower precision and depend heavily on image quality and computational reconstruction algorithms.

Integrating imaging-based metrology with interferometry and spectroscopy can substantially enhance its measurement capability. For example, interferometry-assisted imaging can provide quantitative phase information to complement intensity-based image data, while spectroscopic integration can reveal compositional and material-related parameters. Moreover, embedding AI-driven image reconstruction and data fusion models can transform imaging-based metrology into a perception-oriented framework, enabling real-time, multi-parameter sensing for dynamic and complex environments.

## 4. Spectroscopy-Based Metrology

Spectroscopy-based metrology encompasses a range of optical measurement techniques that extract structural, dimensional, or compositional information by analyzing the wavelength-dependent interaction of light with matter. Representative approaches include absorption and transmission spectroscopy [283], reflection spectroscopy, as well as Raman and fluorescence spectroscopy [284,285,286]. These representative technologies and their corresponding technical characteristics are shown in Table 2. Each method has found specific niches in scientific research and industrial metrology; However, limitations such as low spatial resolution, sensitivity to environmental disturbances, or restrictions on sample material and geometry often constrain their applicability in high-precision, non-contact measurement scenarios.

However, for the measurement of geometrical parameters and surface reconstruction of micro–nano structures, conventional techniques still face limitations in terms of spatial resolution, measurement speed, and adaptability to complex surfaces. Against this backdrop, confocal techniques—including chromatic confocal microscopy [287] and laser confocal microscopy [288]—have emerged as powerful tools, offering high axial resolution and non-contact measurement capabilities. At the same time, scatterometry, a diffraction-based optical metrology technique, has attracted significant attention owing to its advantages in large-area inspection and rapid parameter inversion. This chapter provides a systematic review of these two approaches, highlighting their fundamental principles, recent advances, and application prospects.

### 4.1. Confocal Optical Metrology

#### 4.1.1. Chromatic Confocal Technology

**Figure 5:**
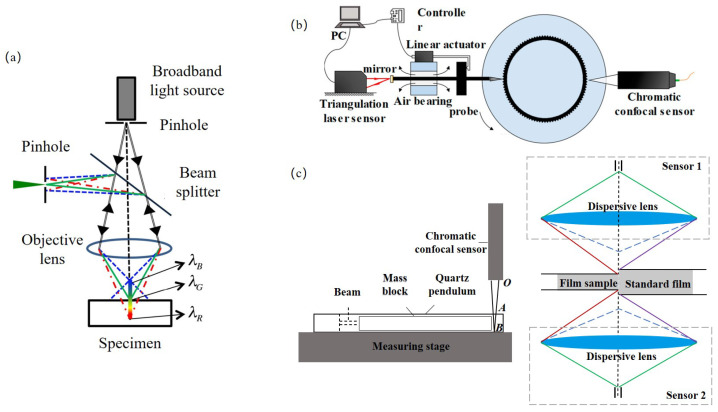
Chromatic confocal technology and its applications: (**a**) CCT principle; (**b**) chromatic confocal sensor for contour measurement; (**c**) chromatic confocal sensor for workpiece thickness measurement.

Chromatic confocal technology (CCT) originates from the confocal optical systems developed by Winston et al. in the 1940s–1950s [289,290], and evolved into a distinct branch with the incorporation of chromatic dispersion principles. Its fundamental concept is to use a dispersive objective to focus different wavelengths at distinct axial positions along the optical axis, thus encoding spatial position into wavelength. By analyzing the focal wavelength of the reflected light from the target surface and referring to a pre-calibrated wavelength–position curve, surface position can be determined with high precision. The basic principle of this method is shown in Figure 5a. It requires no axial scanning, is insensitive to source fluctuations and ambient light, and has become an international standard for surface profilometry. With technological advancements, commercial sensors from companies such as Stil (France), Precitec (Germany), Micro-Epsilon (Germany), ThinkFocus (China), and LightE-Technology (China) now achieve nanometer-scale resolution and linear accuracy, suitable for tilted surfaces and high-frequency measurements.

In applications, chromatic confocal sensors can rapidly acquire surface profiles through lateral scanning, enabling three-dimensional measurement of complex surfaces such as coins and aspheric lenses [291,292,293,294,295], and the obtained profile data can be further used for surface roughness evaluation [296]. With multi-sensor cooperation, multi-dimensional shape reconstruction can be achieved [297,298,299]. This technology has also been integrated into laser processing systems for inline surface inspection [300], gap monitoring in high-speed rotating machinery [301], microsphere position detection in optical traps [302], deformation monitoring of nuclear fuel in radiation environments [303], membrane deformation measurement in filtration [304], dynamic monitoring of vibrating tuning forks [305], and atomic force microscope scanning analysis [306], demonstrating strong adaptability to various objects and environments.

For thickness measurement, chromatic confocal methods include single-sensor step-height measurement, dual-sensor configurations, and multi-interface reflection analysis, applicable to transparent films, tempered glass, and lenses [307,308,309,310,311]. In particular, the full-spectrum fitting method based on thin-film interference theory has achieved nanometer-level thickness precision with micrometer-scale lateral resolution [312]. Overall, chromatic confocal technology combines high precision, strong adaptability, simple integration, and high efficiency, making it a vital tool in surface profile and thickness measurement with broad prospects in industrial inspection and scientific research.

Building upon the diverse applications outlined above, significant research has been devoted to advancing the key components and processing techniques of chromatic confocal systems, aiming to improve axial resolution, expand measurement range, enhance environmental robustness, and enable high-speed multi-point detection. This section reviews recent progress in four critical areas: broadband light sources, dispersive objectives, conjugate pinholes, and spectral detection with signal processing.

Broad spectrum light source

Spectral confocal technology relies on broadband light sources to achieve axial position encoding through wavelength dispersion; thus, the continuity and stability of the light source directly determine the measurement resolution and stability. Early implementations primarily employed incandescent lamps, which were subsequently replaced by halogen lamps, xenon lamps, and light-emitting diodes (LEDs). Among them, white LEDs, with a moderate spectral range (380–760 nm), high optical energy utilization, and ease of integration, have been widely adopted in the development of spectral confocal sensors [313,314]. In contrast, although halogen and xenon lamps can also generate broadband white light, they suffer from significant intensity fluctuations in the visible region, leading to a non-uniform signal-to-noise ratio (SNR) across the full measurement range. Moreover, their long preheating time and short operational lifetime further limit practical applications.

**Figure 6:**
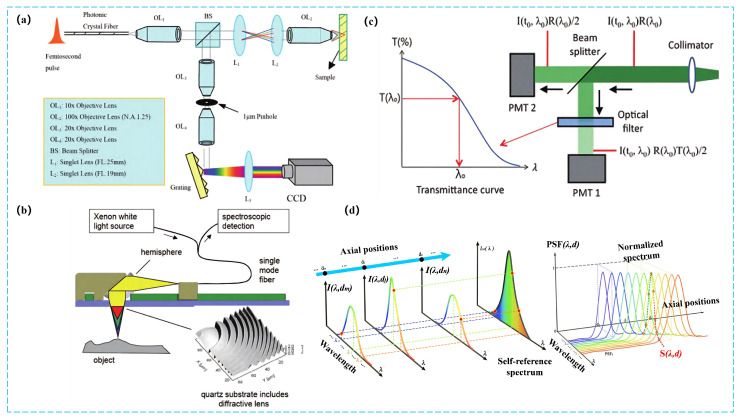
Research progress of chromatic confocal technology: (**a**) light source of chromatic confocal system: PCF device; (**b**) DOE-based chromatic confocal system; (**c**) detection methods of chromatic confocal system; (**d**) normalization strategies of chromatic confocal system: pre-scanned self-reference spectrum.

Based on optical nonlinear effects, pulsed lasers can be modulated to generate broadband supercontinuum (SC) light sources with high brightness and stability, which have emerged as a research hotspot in recent years. As shown in Figure 6a, Shi et al. [315] employed photonic crystal fibers (PCFs) to produce a supercontinuum spanning 350–1750 nm, which, when applied to spectral confocal systems, significantly enhanced illumination efficiency and SNR. Similarly, Minoni et al. [316] reported a 488–1064 nm SC source based on microstructured optical fibers (MOFs), featuring smooth spectral continuity and stable intensity, enabling a displacement measurement repeatability of 0.36%. Liu et al. [317], Johnson et al. [318], and Matsukuma et al. [105] further demonstrated SC sources covering 400–2400 nm for spectral confocal imaging, achieving a broader dispersion range and thereby extending the measurable displacement range.

In addition, Chen et al. [319] utilized a mode-locked femtosecond laser operating in the 1.46–1.64 μm infrared band for spectral confocal displacement measurements, achieving an axial resolution of 30 nm with excellent system stability. However, the dispersion range was limited to 40 μm due to the relatively narrow spectral bandwidth. Overall, both SC sources and mode-locked femtosecond lasers exhibit advantages in spectral stability and measurement performance. Nevertheless, their reliance on high-power lasers results in complex structures, high costs, and potential safety concerns, restricting their application mainly to laboratory environments. By contrast, simple, cost-effective, and stable white LEDs and halogen sources remain the mainstream choice in commercial products.

Dispersive objective lens

The dispersive objective lens is a key component of spectral confocal systems, as it directly determines critical performance parameters such as the measurement range, allowable surface tilt, and light collection efficiency. Current designs can be broadly categorized into refractive optical elements and diffractive optical elements (DOEs).

For refractive optics, Molesini et al. [320] employed a plano-convex lens to introduce dispersion after collimating white light and subsequently focused it with a microscope objective. However, since dispersion was already introduced at the collimation stage and a single lens could not simultaneously optimize both chromatic and other aberrations, the resulting spot quality was limited. Shi et al. [315] and Zhang et al. [321] adopted a pair of thin convex lenses to achieve dispersion after collimation, but the improvement remained modest. Li et al. [322], Niu et al. [323], Liu et al. [324,325], and Shao et al. [326] designed integrated dispersive objectives composed of multiple lenses using Zemax, thereby achieving dispersion while simultaneously optimizing aberrations. Wang et al. [327] and Yang et al. [328] extended the dispersion range to 30 mm using a four-stage cascaded lens group. However, the excessive number of cemented doublets increased system complexity, while random reflections from multiple lens surfaces reduced the overall signal-to-noise ratio (SNR).

For diffractive optics, Dobson et al. [329], Garzón et al. [330], Rayer et al. [331], and Liu et al. [332] employed Fresnel-type DOE lenses to generate dispersion and systematically investigated the influence of diffraction efficiency on the wavelength–position response and resolution. Hillenbrand et al. [333,334] compared the imaging performance of pure DOEs with DOE–lens hybrid objectives, while Jin et al. [335] also proposed hybrid structures. Pruss et al. [336], Fleischle et al. [337], Ruprecht et al. [338], Park et al. [339], and Luecke et al. [340] fabricated DOE lenses with millimeter-scale outer diameters (Figure 6b) for inner-diameter inspection of micro-holes. Hillenbrand et al. [334] further introduced a segmented DOE design that enabled simultaneous displacement measurements at three lateral positions, thereby improving multi-point detection efficiency. Nevertheless, additional reflections and energy loss caused by multiple diffraction orders and double-pass diffraction inherently limit the return efficiency and SNR of DOE-based systems compared with their refractive counterparts.

In summary, refractive optical components remain the mainstream solution in both research and commercialization due to their superior design flexibility, stray-light suppression, and higher return efficiency. By contrast, DOEs offer unique advantages in compactness and array integration, but efficiency and accuracy constraints remain significant bottlenecks to broader adoption.

Conjugate aperture

The conjugate pinhole is the core structure of confocal technology, enabling axial resolution and suppression of out-of-focus light. Its aperture size directly influences both the system resolution and the signal-to-noise ratio (SNR) of the reflected signal. Ruprecht et al. [341] analyzed the impact of aperture size on the performance of spectral confocal sensors, highlighting the trade-off between resolution and light collection efficiency.

To improve measurement efficiency, array-based pinhole designs have been proposed. Tiziani et al. [342,343] and Ang et al. [344] employed a Nipkow disk positioned between the beamsplitter and dispersive objective, enabling the same element to function as both the source and detection pinhole, thereby achieving rapid profilometric scanning. Hwang et al. [345] utilized a rotating disk with pinholes for optical frequency modulation, which significantly enhanced the contrast of three-dimensional imaging. Hillenbrand et al. [346], Chanbai et al. [347], and Hu et al. [348] applied pinhole arrays to split broadband illumination and performed spectral detection through conjugated pinhole arrays, enabling simultaneous measurement of multipoint surface profiles. Cui et al. [349] replaced physical pinholes with two liquid crystal display (LCD) matrices, allowing computer-controlled positioning of measurement points and enhancing the adaptability of the system to complex surface morphologies.

Beyond traditional diaphragm-based pinholes, multimode fibers are also widely used as substitutes for conjugate pinholes. With core diameters ranging from tens to hundreds of micrometers, multimode fibers simplify the optical configuration, reduce alignment complexity, and offer the advantages of low transmission loss and high stability. Luo et al. [350], Chen et al. [319], Bai et al. [314], and Chen et al. [351] introduced multimode fibers or multimode fiber couplers into spectral confocal systems, which not only preserved the confocal effect but also facilitated modular design and long-distance signal transmission.

In summary, pinhole arrays enable rapid multipoint imaging and improved measurement efficiency, but their resolution is limited by pinhole spacing and scattering interference. Multimode fibers, in contrast, are well suited for high-precision single-point detection. Although less efficient, they offer unique advantages in modularity and long-distance measurement.

Spectral detection and signal processing

In spectral confocal systems, the axial displacement is inferred from the focal wavelength of the reflected light, making spectral detection and signal processing critical components. The conventional approach involves dispersing polychromatic light into monochromatic components using dispersive elements such as prisms or gratings, with photodetectors recording the intensity of each wavelength. Molesini et al. [320] and Shi et al. [352] employed triangular prisms for spectral analysis, while Shi et al. [315] also utilized a diffraction grating. Taphanel et al. [353,354] adopted a color camera for coarse spectral measurements. The commercial spectrometers used by Minoni et al. [316] and Luo et al. [350] similarly relied on prisms or gratings as their core dispersive components.

To enhance detection speed, Kim et al. [355] exploited the relationship between wavelength and filter transmittance, calibrating axial position directly from transmittance. This method required only two photomultiplier tubes and avoided temporal integration, achieving measurement rates up to hundreds of MHz (as illustrated in Figure 6c). Chen et al. [356] used dual-color CCDs to acquire differential signals corresponding to positions instead of explicit wavelengths, thereby improving detection efficiency. However, both methods are limited to specific wavelength bands and constrained by relatively low spectral resolution, restricting further improvements in displacement accuracy.

To address the effects of source spectrum inconsistency and spectral jitter, various normalization schemes for reflected spectra have been proposed. For example, Luo et al. [350] directly adopted the source spectrum as a reference, Minoni et al. [316] used the reflected spectrum obtained without the dispersive objective as reference, Yu et al. [357] applied focal wavelength compensation for surfaces of different colors, and Bai et al. [358] introduced a pre-scanning self-referencing method, which improved the universality of the focal wavelength–position response curve (as shown in Figure 6d).

In terms of peak-search algorithms, Molesini et al. [320] adopted parabolic fitting, Shi et al. [352] directly used the maximum intensity, and Tan et al. [359] applied a sinc^2^ curve fitting. Ruprecht et al. [360] and Deng et al. [361] implemented centroid-based methods, which offer high computational efficiency. Niu et al. [362] and Luo et al. [350] compared centroid, Gaussian fitting, thresholded centroid, and thresholded Gaussian fitting approaches, concluding that Gaussian fitting achieves the highest accuracy while centroid methods provide the best efficiency. Chen et al. [351] and Lu et al. [363] further proposed modified differential fitting and mean-shift algorithms, achieving accuracy comparable to Gaussian fitting with significantly improved computational efficiency.

Overall, spectral detection and signal processing are progressing toward higher speed, broader applicability, and greater intelligence. In the future, integration with machine learning techniques may enable adaptive peak-searching and high-precision displacement reconstruction under complex operating conditions.

#### 4.1.2. Confocal Laser Scanning Microscopy

**Figure 7:**
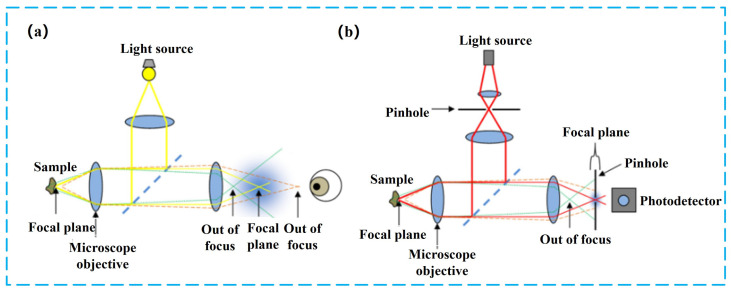
Comparison between (**a**) conventional optical microscopy and (**b**) confocal laser scanning microscopy.

Laser Confocal Microscopy (LCM), also referred to as Confocal Laser Scanning Microscopy (CLSM), is a high-resolution three-dimensional imaging and measurement technique based on optical focusing and spatial filtering. Compared with conventional optical microscopy (Figure 7), its core advantage lies in the use of a confocal pinhole to effectively suppress out-of-focus light, thereby enabling the acquisition of high-contrast, high-resolution images and three-dimensional reconstructions [364]. In an CLSM system, a point light source (typically a laser) illuminates the sample surface. The reflected or transmitted light is collected by the objective lens and directed toward a pinhole placed in front of the detector. The pinhole only permits light from the focal plane to pass through while blocking scattered light from out-of-focus regions. As a result, the system produces optical sectioning images with superior spatial resolution. By sequential point scanning and signal reconstruction, three-dimensional imaging and surface topography measurement of the sample can be achieved.

Since its introduction, CLSM has evolved into a powerful imaging modality with broad applications across biology, materials science, nanotechnology, and clinical diagnostics. The early foundation of CLSM was established by Sheppard and Wilson, who demonstrated that spatial filtering with a pinhole could effectively suppress out-of-focus light and achieve optical sectioning with sub-micron resolution, far surpassing conventional wide-field microscopy [365]. Pawley and collaborators further refined laser scanning configurations, enabling accurate three-dimensional reconstructions in biological tissues [366].

In biological imaging, fluorescence-based CLSM marked a significant breakthrough when Tsien and coworkers combined fluorescent dyes with confocal detection, providing real-time visualization of live-cell dynamics with high spatial resolution [367]. Later, Denk et al. pioneered the integration of multiphoton excitation with CLSM, which greatly improved penetration depth in thick tissues while reducing scattering and phototoxicity [368]. Computational strategies such as deconvolution and adaptive optics were subsequently incorporated, allowing aberration correction and further enhancing image sharpness [369].

In materials science, Schubert et al. demonstrated that CLSM could probe thin-film morphology and characterize surface roughness with depth selectivity, establishing its value in semiconductor and coating analysis [370]. More recent progress in nanophotonics has enabled CLSM to be combined with plasmonic substrates for surface-enhanced chemical sensing and nanomaterial characterization, thereby extending its applications to nanoscale metrology [371].

In the clinical domain, CLSM has been successfully translated into dermatological diagnostics. Rajadhyaksha et al. developed handheld CLSM devices for in vivo skin imaging, achieving cellular-level resolution for non-invasive detection of basal cell carcinoma and melanoma [372]. Clinical studies have demonstrated diagnostic sensitivities and specificities exceeding 90%, confirming its utility as a non-invasive adjunct to histopathology in skin cancer management [373,374].

Overall, CLSM has progressed from a fundamental optical innovation to a versatile and multidisciplinary imaging platform. With continuing advances in laser sources, detection schemes, and computational integration, CLSM is expected to further expand its role in nanometrology, biomedical imaging, and precision clinical diagnostics.

Differential Confocal Microscopy

Differential confocal microscopy (DCM) is an optical technique based on the confocal principle, enabling high-precision axial localization and surface profilometry [375]. By employing a differential detection mechanism, DCM reconstructs the axial response characteristics of conventional confocal microscopy, with its core lying in the construction of a dual-channel differential optical path. In its basic configuration, two detectors are symmetrically placed before and after the confocal plane to capture the reflected intensity from the sample. When the sample is exactly at the confocal plane, the two detectors receive equal intensities; as the sample moves axially, the detector responses change in opposite directions. By calculating the difference (or normalized difference) between the two signals, a response curve with monotonicity and a zero-crossing feature is obtained, enabling nanometer-scale axial localization accuracy [376].

Compared with conventional confocal imaging, DCM not only significantly improves axial resolution but also demonstrates strong noise immunity and a wide linear measurement range. It has thus been widely applied in microstructure profile reconstruction, thickness evaluation, and precision displacement control for advanced metrology. In 2000, Wang et al. first introduced the concept of DCM, achieving an axial resolution of 2 nm (with a measurement range of 100 μm). This established the fundamental differential detection structure, offering both absolute measurement and focus-tracking capability [377]. Over the subsequent two decades, researchers have continuously refined DCM techniques: Sun et al. [378] developed axial high-resolution DCM (AHDCM), in which sample position is determined through energy curves from three optical paths; Yun et al. [379,380] introduced D-shaped pupil DCM (DDCM), achieving 5 nm axial resolution at a 3.1 mm working distance with a threefold increase in imaging speed; Zou et al. [381] combined D-shaped pupils with radially birefringent filters to improve lateral resolution; Wang et al. [382] applied radially polarized illumination and pupil filtering for high-precision measurements.

In practice, DCM faces two major challenges: (i) test surfaces are often non-ideal planes [383]; and (ii) when employed as a profilometric probe, a trade-off exists between accuracy and scanning efficiency [384]. Although high-NA objectives can enhance axial resolution [385], sloped surfaces reduce optical energy collection, lowering SNR and resolution while introducing aberrations and artifacts. Mauch et al. [386] confirmed focal shift phenomena when using microscope objectives in freeform surface measurements. To address this, Wang et al. [387] proposed a slope measurement system and algorithm based on dual cylindrical lenses, enabling simultaneous acquisition of both spatial position and slope at the sample surface. For slope-related errors, Cacace et al. [388] employed position-sensitive detectors (PSDs) in 2009, but did not account for increased localization error and reduced resolution at large slopes. More recently, Wang et al. [376] introduced a Pearson correlation coefficient (PCC) compensation strategy based on peak clustering, which was applied to a DCM probe system. By increasing the sampling interval, this method enhanced scanning efficiency while maintaining preset accuracy, offered greater tolerance to localization errors, and compensated for the effects of non-normal incidence, thereby improving 3D measurement accuracy and system robustness.

With its superior axial resolution and structural flexibility, DCM offers unique advantages in high-precision surface metrology. Through the integration of novel structural designs, adaptive scanning strategies, and advanced signal processing algorithms, this technique continues to overcome the limitations of traditional confocal approaches, making it increasingly applicable to diverse micro- and nanometrology scenarios. Looking ahead, the combination of DCM with artificial intelligence-based image processing, active feedback control, and multimodal integration technologies is expected to further enhance imaging speed, system intelligence, and adaptability, establishing DCM as a key component of next-generation hybrid optical metrology systems.

Overall, research progress in these five areas has significantly expanded the capabilities of chromatic confocal technology, enabling higher resolution, larger ranges, faster acquisition rates, and broader application adaptability, thereby reinforcing its role as a critical tool in precision metrology and industrial inspection. Despite significant progress, the technology still faces three core challenges: designing dispersive optics with ultra-long measurement ranges (>10 mm), achieving dynamic measurement stability for highly reflective and multilayer transparent materials, and unifying precision across scales from nanometers to millimeters. Future development efforts are focused on the following areas: (1) integration of intelligent algorithms; (2) multimodal technology integration; (3) miniaturization and industrialization.

### 4.2. Optical Scatterometry

Optical scatterometry is a non-destructive metrology technique based on the interaction between light and periodic nanostructures. Its fundamental principle involves analyzing the intensity, polarization state, or phase variations of diffracted light to invert the geometric parameters of the nanostructures, such as critical dimension (CD), height (H), and sidewall angle (SWA). The Rigorous Coupled-Wave Analysis (RCWA) method, proposed by Moharam and Gaylord in the early 1980s [389], provides a solid mathematical foundation for this technique. RCWA solves Maxwell’s equations to establish a quantitative mapping between the structural geometric parameters and the optical diffraction response. Owing to its high throughput, non-contact nature, and low cost, optical scatterometry has become one of the core online metrology tools in semiconductor manufacturing.

Essentially, optical scatterometry is a model-based metrology (MBM) approach [390], and its measurement process represents a typical inverse problem. Its success relies on two critical technical aspects: first, accurate forward modeling of optical properties and efficient solution methods; second, robust algorithms for the inversion of geometric feature parameters. Therefore, advancing effective optical modeling and simulation methods for nanostructures, as well as developing fast and robust inversion algorithms for geometric parameters, has become a research focus in recent years to enhance measurement accuracy and expand the applicability of optical scatterometry.

Compared with CD-SEM and CD-AFM, optical scatterometry based on spectroscopic ellipsometry offers advantages such as high measurement speed, low cost, non-destructive operation, and ease of online integration. Beyond its widespread use in characterizing the optical properties and film thickness of thin-film materials [391,392], spectroscopic ellipsometry began to be applied around the year 2000 for critical dimension (CD) measurements of subwavelength nanostructures [393,394,395,396].

Spectroscopic ellipsometry (SE) measures the change in polarization of reflected light, expressed by the amplitude ratio (Ψ) and phase difference (Δ) between p- and s-polarized components:

(11) $$\tan\psi e^{i\Delta }=\frac{r_{p}}{r_{s}}$$

Here, $r_{p}$ and $r_{s}$ denote the complex reflection coefficients for p-polarized and s-polarized light, respectively. Using this relationship, one can invert the material’s optical constants (refractive index *n* and extinction coefficient *k*) or the thickness of thin films.

Spectroscopic ellipsometry (SE) is widely employed for thin-film thickness measurements in solar cells and microelectronic devices. It has also been applied in early optical critical dimension (OCD) measurements of nanogratings, although its accuracy was limited by the insufficient dimensionality of the measured parameters.

SE exhibits extremely high sensitivity to thin-film thickness, with measurement precision reaching the angstrom (Å) scale. For example, Fujitsu Laboratories integrated SE into an atomic layer deposition (ALD) system to monitor film growth rates in real time, achieving control precision of ±0.1 nm [397]. SE is well suited for rapid characterization of isotropic thin films, such as SiO_2_ and photoresists. However, as it measures only the two parameters Ψ and Δ, its sensitivity to nanostructures with complex cross-sectional morphologies (e.g., gratings with slanted sidewalls) is limited. In addition, the measurement spot size is relatively large (typically >100 μm), making it difficult to resolve structural variations in small regions.

#### 4.2.1. Mueller Matrix Ellipsometer

To overcome the limited information content of conventional spectroscopic ellipsometry (SE), Mueller matrix ellipsometry (MME) measures the complete 4×4 Mueller matrix, providing 16 independent parameters that fully characterize the sample’s polarization response, including anisotropic effects and depolarization phenomena [392]. Its implementation relies on modulating multiple incident polarization states and analyzing the corresponding scattered light. Since its inception, generalized ellipsometers have been widely applied in the characterization of anisotropic materials and in the measurement of geometric parameters of nanostructures [398,399].

At the Interface and Thin Film Physics Laboratory of CNRS-LPICM, Novikova and colleagues have been investigating the use of Mueller matrix ellipsometry for grating critical dimension (CD) and overlay error measurements since 2005 [400]. They highlighted that MME provides richer information than conventional spectroscopic ellipsometry and emphasized the importance of varying the azimuthal angle to achieve conical diffraction [401]. In 2007, Schuster et al. at the University of Stuttgart proposed introducing azimuthal scanning (ϕ-scan) into conventional spectroscopic ellipsometry [402]. By recording the Mueller matrix as a function of azimuth at a fixed wavelength, simulations showed enhanced sensitivity to two-dimensional asymmetric hole arrays compared with wavelength scanning (λ-scan) or incident angle scanning (θ-scan), particularly for matrix elements m32 and m43. More recently, Nanometrics reported that MME exhibits high sensitivity to both direction and magnitude of grating overlay errors and asymmetry, whereas conventional SE is largely insensitive [403]. They also suggested that analyzing the relationship between specific matrix elements and structural features could enable rapid measurements.

Collins proposed a dual-rotating compensator configuration in 1999 [404], in which two compensators rotate synchronously at a frequency ratio of 5:3 to efficiently acquire the full Mueller matrix. This design covers a broad spectral range of 193–1000 nm and achieves calibration accuracy better than 0.005°. Liu et al. extended this design for generalized ellipsometers, implementing a dual-rotating compensator architecture with three degrees of freedom—wavelength, incident angle, and azimuth—enabling the acquisition of all 16 parameters in a single measurement. The resulting Mueller matrix element (MME) showed threefold higher sensitivity to line-edge roughness (LER) than SE, making it suitable for critical dimension measurements at sub-10 nm nodes [405]. Garcia-Caurel et al. confirmed that MME can accurately analyze optical anisotropy in complex samples such as liquid crystals and crystals [406].

#### 4.2.2. Imaging Ellipsometer

**Figure 8:**
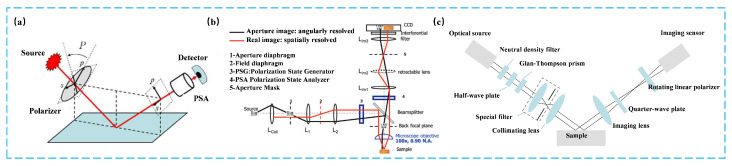
General scheme of ellipsometer: (**a**) a standard ellipsometer; (**b**) a imaging–conoscopic Mueller polarimeter; (**c**) a three-step phase shift imaging ellipsometer.

Mueller Matrix Imaging Ellipsometry (MMIE) combines microscopic imaging with Mueller matrix ellipsometry (MME) to effectively overcome the spatial resolution and efficiency limitations of conventional scatterometry. In 2015, Liu et al. at Huazhong University of Science and Technology integrated MME with microscopic imaging, developing a Mueller matrix imaging ellipsometer. Compared with traditional spectroscopic ellipsometers, this system provides richer information, higher sensitivity, and the ability to vary the azimuthal angle to achieve conical diffraction [401], enabling large-area, rapid, and accurate measurements of nanoscale structural parameters. The system employs dual-rotating compensators, offering broad spectral measurement capability while simplifying system calibration and data processing. This approach not only retains the advantages of conventional MME but also incorporates the high spatial resolution of microscopic imaging, covering a spectral range of 190–1000 nm. In 2016, the group completed the development of China’s first high-precision, broadband Mueller matrix imaging ellipsometer, with the ellipsometric imaging configuration illustrated in Figure 8 [407,408].

In 2018, Hanyang University in South Korea proposed a dual-reflection ellipsometric imaging system [409], which generates differential images by comparing the polarization state changes between a reference and a test sample. This approach eliminates the need for zeroing, achieves measurement rates up to the camera’s maximum frame rate, reduces human error, and does not require uniform illumination, allowing real-time detection of surface defects and contamination. In 2019, Tohoku University in Japan developed a three-step phase-shifting ellipsometric imaging technique [410,411], combining a quarter-wave plate (QWP) and a rotating linear polarizer (RLP) to implement phase shifts. Images are captured at three equally spaced azimuth angles during constant-speed rotation, enabling nanoscale material thickness measurements.

Also in 2019, Huazhong University of Science and Technology developed a vertically oriented, liquid-crystal-based Mueller matrix imaging ellipsometer [412], addressing the limited depth of field and narrow field of view associated with oblique imaging, and achieving high-resolution, wide-field measurements suitable for nanoscale thin-film geometric characterization. In 2018, Chosun University in South Korea proposed a large-area spectroscopic imaging ellipsometer [413,414], integrating a broadband light source with an imaging spectrometer (400–800 nm) and expanding the beam to 30 mm before low-magnification lens imaging. This system captures spectral–spatial intensity maps of polarization variations, enabling large-area thin-film thickness profiling with lateral resolution down to 4 μm, thus advancing ellipsometric imaging toward wide-field, broadband applications.

### 4.3. Summary

Spectroscopy-based metrology determines physical or chemical properties by analyzing the interaction between light and matter across different wavelengths. Techniques such as absorption, reflection, fluorescence, and Raman spectroscopy are widely used for material identification, thin-film measurement, and microstructure characterization. The method provides excellent sensitivity to material composition and enables non-destructive, label-free analysis. However, its spatial resolution and measurement speed are typically lower than those of imaging or interferometric approaches, and the complexity of spectral data processing often limits its real-time performance.

Combining spectroscopy with imaging or interferometric modalities allows for the simultaneous capture of both structural and compositional information, enhancing the overall diagnostic capability of optical systems. For instance, integrating spectroscopy with interferometry can improve phase stability through wavelength tuning, while coupling with imaging enables spatially resolved spectral mapping. The integration of broadband light sources, computational spectroscopy, and machine-learning-driven spectral analysis represents a promising direction toward intelligent hybrid metrology systems capable of high-precision, multi-dimensional perception.

## 5. Hybrid & Frontier Metrology

The emergence of hybrid and frontier metrology signifies a paradigm shift from precision-oriented measurement to perception-oriented sensing. By incorporating computational intelligence, multimodal fusion, and real-time decision-making capabilities, these systems bridge the gap between traditional metrology and perceptual understanding, forming the core of next-generation optical technologies.

### 5.1. Hyperspectral Imaging Metrology

Hyperspectral imaging (HSI) integrates imaging and spectroscopic measurement into a single system. By acquiring high spectral resolution samples of the target’s reflectance or radiance across tens to hundreds of contiguous narrow spectral bands (typically spanning the visible to near-infrared range of 0.4–2.5 μm), it enables the identification of subtle material characteristics. The basic principle is to use prisms, gratings, or tunable filters to disperse light by wavelength, and then employ a two-dimensional detector to acquire spectral and spatial information simultaneously. System architectures mainly include push-broom, step-scan, snapshot, and point-scanning modes, among which the push-broom configuration is widely applied in remote sensing and industrial domains due to its high spectral resolution and broad coverage [415].

In recent years, rapid advances in optical device fabrication and data processing algorithms have driven significant breakthroughs in HSI with respect to spatial resolution, imaging speed, and intelligent data processing. On one hand, compact HSI systems based on distributed gratings and micro/nano-optical components have emerged, greatly improving miniaturization and portability to meet the needs of applications such as industrial in-line inspection and handheld measurement [416,417,418]. For example, compact hyperspectral cameras based on on-chip thin-film filters and quantum dot materials (e.g., Specim IQ) have been deployed in industrial online inspection and handheld terminal devices [419]. On the other hand, novel HSI methods that integrate computational imaging with compressed sensing theory have become a research frontier: by combining optical encoding with reconstruction algorithms, these approaches dramatically reduce data acquisition volume and transmission load, enabling efficient, real-time hyperspectral data acquisition [420]. Furthermore, deep learning-driven spectral feature extraction and object recognition techniques are continuously advancing, significantly enhancing HSI’s detection and classification capabilities in complex scenarios. Convolutional neural networks (CNNs), transformer models, and other architectures have been widely applied to hyperspectral image feature extraction, denoising, and super-resolution reconstruction, demonstrating outstanding performance in applications such as surface defect detection in precision manufacturing and quality assessment of semiconductor materials [421,422].

In agriculture and food inspection, HSI has been widely used for crop growth monitoring, early-stage pest and disease diagnosis, and quality grading of agricultural products. By mounting hyperspectral sensors on UAVs, precision agricultural management can be carried out at the field scale, improving the accuracy of crop classification and disease detection. In the food industry, HSI has been applied to egg freshness prediction, as well as classification and inspection of agricultural products such as pine nuts and coffee beans; its non-destructive, full-field measurement capability has made it an important tool in modern food quality control [423]. In industrial non-destructive testing and intelligent manufacturing, HSI has shown great potential for detecting adhesive residue, surface cracks, and latent defects in carbon fiber composite production, effectively supporting the establishment of automated inspection and quality control systems [424]. In the field of medical diagnosis, with the establishment of hyperspectral brain imaging databases and advances in real-time data annotation techniques, HSI is increasingly being applied in intraoperative assistance for brain tumor surgery, enabling real-time tumor boundary recognition and visual guidance based on spectral differences between tissues [425]. Moreover, with the growing demand for global environmental monitoring, hyperspectral remote-sensing satellites such as Germany’s EnMAP and India’s Pixxel commercial satellite projects have expanded HSI applications into broader domains including terrestrial ecosystem monitoring, mineral resource exploration, and atmospheric environment observation, pushing the technology from laboratories into diverse industrial fields [426,427]. Waste recycling and intelligent sorting are also emerging application areas, where HSI’s high sensitivity to the spectral characteristics of different plastics and solid waste materials is reshaping the smart recycling industry chain, providing strong support for green manufacturing and sustainable development [428].

Despite its advantage of high resolution, HSI still faces challenges such as data redundancy, high cost, and lack of standardization. Future technological developments will focus on sensor miniaturization, deep integration with AI, multi-modal sensing, and standardization efforts. With advances in MEMS technology and quantum dot materials, HSI is expected to further penetrate consumer electronics and smart devices, while breakthroughs in quantum sensing may enable single-molecule-level spectral detection, opening new fields for nanoscale material analysis [429,430].

Overall, hyperspectral imaging technology is evolving toward the “three-high” direction of high-spectral, high-spatial, and high-temporal resolution, and is progressively integrating with multi-modal detection methods such as Raman spectroscopy, photoacoustic imaging, and infrared thermography. Its application boundaries in precision manufacturing, life sciences, resource and environmental fields, and intelligent manufacturing are continually expanding. In the future, with the deep integration of edge computing and artificial intelligence into hyperspectral data processing, HSI will achieve an integrated closed-loop workflow from high-speed data acquisition to real-time intelligent analysis, facilitating the widespread adoption of optical metrology methods in next-generation advanced manufacturing systems.

From an integration perspective, hyperspectral imaging serves as a natural bridge between imaging-based and spectroscopy-based metrology. Its capability to capture both spatial and spectral dimensions enables multimodal fusion with complementary techniques such as Raman spectroscopy, interferometry, and infrared thermography. The main challenges include balancing spectral and spatial resolution, managing large data volumes, and maintaining cross-modal calibration. Future developments will emphasize miniaturized sensors, AI-assisted data reconstruction, and edge-computing-based fusion pipelines that enable real-time analysis and interpretation. Through such integration, hyperspectral imaging will evolve from a stand-alone modality toward a core component of intelligent, perception-oriented optical metrology systems.

### 5.2. Optical Vortex-Based Metrology

Optical vortices, as light beams carrying orbital angular momentum (OAM), have attracted considerable attention in recent years within the field of high-precision optical metrology. Compared with conventional Gaussian beams, they possess more intricate phase structures and exhibit unique advantages in applications such as angular displacement sensing, micro-force measurement, and phase imaging. First introduced by Allen et al., optical vortices are characterized by a helical phase term expressed as exp(ilϕ), where *l* denotes the topological charge, representing the quantum number of the orbital angular momentum carried by the beam [431]. This helical phase distribution results in a hollow dark core at the beam center, endowing the light field with both spatial tunability and exceptional sensitivity to minute angular and displacement variations, thereby establishing it as a powerful tool in contemporary optical metrology.

From a fundamental perspective, the metrological capability of optical vortices primarily arises from their distinctive response to rotationally symmetric perturbations. Due to the strict orthogonality among vortex beams with different topological charges, system measurements can be achieved by detecting variations in interference fringes, OAM mode transitions, or wavefront distortions to infer rotational, deformation, or displacement information of the target. For example, when an optical vortex propagates through a slightly rotated object or phase plate, its phase distribution undergoes a characteristic distortion that can be precisely captured through interferometric techniques, enabling high-accuracy angular sensing [432,433]. In optical trapping systems, the orbital angular momentum of vortex beams can impart nanoscale torques for cellular manipulation [434]. Furthermore, in multimode OAM systems, vortex beams of different orders are exploited for parallel encoding, thereby extending the channel capacity of optical communication systems [435].

In terms of implementation, optical vortex metrology has evolved along multiple technological pathways. On the generation side, diverse methods have been developed, including spatial light modulators (SLMs), spiral phase plates, metasurfaces, and integrated optical devices, all capable of producing OAM beams of varying orders [436,437,438,439]. On the detection side, various schemes based on interferometry, Fourier mode decomposition, and machine learning have been introduced, leading to significant improvements in the accuracy of OAM state recognition and retrieval [440,441,442]. Vortex-based interferometers have also emerged as a research hotspot. For instance, OAM interferometers modified from Mach–Zehnder configurations have demonstrated measurement precision at the nanometer scale for linear displacement and at the picoradian scale for angular displacement [443]. In surface imaging, the helical phase of vortex beams can serve as a wavefront probe, enhancing both the sensitivity and resolution of surface profile and roughness characterization [444,445].

Despite their promising prospects, several challenges remain in the practical deployment of optical vortex metrology. The generation and control of OAM beams demand stringent experimental conditions and high-precision components, with devices such as SLMs and phase plates being costly and system architectures often complex. Moreover, vortex beams are susceptible to turbulence and system-induced errors during propagation, which can compromise stability. In addition, the demultiplexing and recognition of higher-order OAM modes still rely on sophisticated algorithms and multidimensional detection architectures, limiting real-time performance and portability. Nevertheless, owing to their non-contact nature, high sensitivity, and capacity to encode high-dimensional information, optical vortex metrology has become an essential complement to existing optical measurement techniques. It holds significant potential for applications in precision manufacturing, biomedical imaging, and emerging quantum sensing technologies [446].

Integrating vortex-based metrology with other optical modalities—such as interferometry and holography—can provide complementary phase, angular, and structural information, extending its applicability in precision manufacturing and biomedical imaging. However, challenges remain in maintaining OAM stability under turbulence, simplifying high-order mode control, and achieving real-time demultiplexing for dynamic measurements. Future research may focus on adaptive wavefront correction, learning-based OAM state recognition, and integrated photonic devices for compact, vibration-tolerant implementations. These advances will enhance robustness and scalability, supporting the deployment of vortex-assisted hybrid metrology in complex industrial environments.

### 5.3. AI-Assisted Optical Metrology

In recent years, artificial intelligence (AI) has demonstrated disruptive potential in the field of optical metrology, offering new pathways toward achieving higher accuracy, greater efficiency, and enhanced adaptability. Traditional optical metrology techniques have largely relied on rigorous physical modeling, such as Rigorous Coupled-Wave Analysis (RCWA), the Finite-Difference Time-Domain method (FDTD), or phase retrieval algorithms. While these approaches are powerful, they often involve heavy computational loads and exhibit high sensitivity to noise. By contrast, AI methods based on machine learning (ML) and deep learning (DL) provide data-driven alternatives that directly map optical signals to target metrological parameters, thereby substantially reducing computational costs and improving robustness under experimental uncertainties [447,448,449].

One of the most significant applications of AI in optical metrology lies in solving inverse problems, namely reconstructing structural or material parameters from measured optical responses. Neural networks trained on simulated or experimental datasets enable rapid extraction of nanoscale parameters, thereby overcoming the bottlenecks of traditional iterative optimization techniques. For instance, convolutional neural networks (CNNs) have been employed in scatterometry for estimating critical dimensions and sidewall angles, as well as in nanoscale tomographic modeling, demonstrating superior accuracy and noise tolerance compared with conventional regression methods [450,451,452,453]. Furthermore, physics-informed neural networks (PINNs), which embed Maxwell’s equations into the learning process, have shown enhanced generalization capabilities beyond the training domain [454,455].

Another rapidly advancing direction is AI-enabled image-based optical metrology, particularly in microscopy, interferometry, and holography [456,457,458]. Deep learning models have been widely applied to phase retrieval, holographic reconstruction denoising, and super-resolution imaging, effectively overcoming the diffraction limit. In digital holographic microscopy, AI-driven phase retrieval methods eliminate the need for complex iterative algorithms, enabling real-time imaging of biological specimens and nanostructures [459]. Similarly, in interferometric profilometry, generative adversarial networks (GANs) have been utilized for speckle noise suppression and high-dynamic-range reconstruction, significantly improving measurement accuracy under challenging conditions [460,461].

AI has also facilitated the development of adaptive and autonomous optical metrology systems. Reinforcement learning (RL) has been introduced to optimize measurement configurations, such as incident angle, polarization state, wavelength selection, and sensor alignment, enabling systems to autonomously adapt to varying sample properties [462,463,464]. This adaptive paradigm not only enhances efficiency but also broadens the applicability of optical metrology in complex industrial environments, particularly in scenarios characterized by high sample diversity and stringent speed requirements.

Nevertheless, the application of AI in optical metrology faces several challenges. Its “black-box” nature has raised concerns, particularly in high-stakes applications such as semiconductor manufacturing and biomedical diagnostics, where model interpretability and reliability remain critical. Moreover, the dependence on large, representative datasets limits its scalability, especially in cases where experimental data acquisition is costly. To address these issues, hybrid approaches combining physical modeling with AI—such as physics-guided deep learning—have emerged as effective strategies, ensuring both predictive accuracy and physical interpretability [465].

In summary, AI-assisted optical metrology is driving a paradigm shift from model-driven to data-driven methodologies. Current studies have already demonstrated notable advantages in terms of speed, precision, and adaptability. Future research is expected to focus on developing interpretable AI frameworks, enhancing cross-domain transferability, and establishing standardized validation benchmarks. As AI technologies continue to mature, they are poised to become the central driving force behind next-generation optical metrology systems, meeting the stringent demands of advanced manufacturing, materials science, and biomedical imaging.

Beyond standalone applications, AI also plays a central role in connecting heterogeneous metrology modalities into unified, perception-oriented frameworks. Key challenges lie in ensuring cross-domain data consistency, model interpretability, and generalization under unseen conditions. Physics-informed learning and multimodal training strategies—integrating interferometric phase, spectral, and imaging data—offer promising routes toward robust fusion models. Furthermore, embedding AI algorithms into optical hardware platforms such as FPGA or neuromorphic processors can enable real-time adaptive metrology. These developments represent a crucial step toward autonomous optical measurement systems capable of continuous optimization and intelligent decision-making.

## 6. Conclusions and Perspective

Optical metrology and sensing constitute a critical field that has already played, and will continue to play, a pivotal role in science and technology. Given its increasing impact, it is necessary to provide a comprehensive overview—from fundamental principles to advanced techniques and future perspectives. This work systematically reviews multimodal approaches in optical metrology and sensing, and proposes a four-category framework consisting of interferometry, imaging, spectroscopy, and hybrid & frontier technologies. For each category, a three-dimensional analytical model—principle, scenario adaptation, and latest developments—is constructed to map the technological evolution and interdisciplinary connections. The review covers applications such as industrial inspection (e.g., nanoscale defect detection in semiconductors) and consumer electronics (e.g., AR/VR spatial tracking), with the aim of providing guidance for technology selection.

Looking ahead, optical metrology exhibits several development trends.

(1) Hardware innovation aims to realize compact, stable, and high-performance systems within physical and environmental limits. While progress in miniaturized light sources, EUV optics, and metasurface components has been remarkable, challenges remain in scaling integration without compromising stability or yield. Future work will focus on CMOS–MEMS on-chip integration, high-uniformity EUV gratings, noise-resilient quantum emitters, and reconfigurable nanophotonic devices to achieve scalable and reliable hardware foundations.

(2) Fusion of fundamental and emerging paradigms targets unified multimodal frameworks that combine interferometric, imaging, spectroscopic, and quantum modalities for multidimensional sensing. Core challenges include cross-domain calibration, temporal synchronization, and the interpretability of AI-driven fusion models. Promising directions include hyperspectral imaging with joint AI modeling, quantum-enhanced sensing, frequency-comb-based references, and wide-FOV AR/VR systems. Establishing unified standards and physically interpretable fusion models will be crucial for dependable integration.

(3) Signal processing and intelligent analysis seek to transform optical data into actionable insights through real-time, automated interpretation. Despite significant advances in AI-assisted inspection, adaptive multi-sensor fusion, and all-optical processing, issues of data reliability, interpretability, and resource efficiency persist. Future developments will emphasize physics-informed learning, edge–cloud collaboration, and co-design of optical–electronic architectures to ensure accurate, traceable, and energy-efficient measurement.

In summary, the future of optical metrology lies in the convergence of precision, intelligence, and integration. Progress in low-noise quantum sources, stable EUV systems, and intelligent data architectures will drive the transition from measurement-oriented systems to perception-oriented optical metrology capable of understanding and adapting to complex environments.

## Figures and Tables

**Table 1:** Classification of optical measurement techniques across multiple spatial scales.

Scale	Accuracy	Measurement Range	Technique	Typical Application
Nanometer	±0.1 nm	0.1–1 mm	White-light interferometry	Wafer surface topography inspection
Nanometer	±1 nm	0.01–0.5 mm	Fizeau interferometry	Optical component surface metrology
Micrometer	0.2 μm	0.1–5 mm	Laser confocal microscopy	Reverse engineering of precision parts
Micrometer	0.1 μm	1–10 mm	Spectral confocal microscopy	Thickness measurement of transparent materials
Micrometer	±0.5 μm	0.05–2 mm	Structured light (sinusoidal fringe)	High-reflectivity surface inspection
Millimeter	±5 μm	50–500 mm	Structured light (speckle encoding)	3D reconstruction of automotive bodies
Millimeter	±0.1 mm	3–7 cm	Laser triangulation	Dimensional inspection of industrial parts
Centimeter	±3 mm	0.1–300 m	LiDAR (dToF)	Large-scale 3D industrial mapping
Centimeter	±1 cm	1–10 m	Stereo vision	Kinect motion capture
Decimeter	±5 cm	1–50 m	iToF phase ranging	VR gesture interaction
Meter	±0.1%	1–100 m	FMCW coherent ranging	Autonomous driving obstacle detection
Cross-scale	Pixel level	0.1–100 m	Computational imaging	Imaging through scattering media
Cross-scale	Photon level	1–1000 m	Quantum imaging	Single-photon night vision systems
Special scale	±10 nm	1–100 μm	Microscopic imaging	3D reconstruction of biological cells

**Table 2:** Common Spectral Detection Techniques.

Category	Typical Methods	Measurement Principle	Characteristics and Limitations
Absorption/ Transmission	UV–Vis, NIR spectroscopy	Measures light absorption or transmission at specific wavelengths	Offers fast data acquisition and simple setup; however, spatial resolution is limited for material composition analysis.
Reflection Spectroscopy	White-light reflectance, RIFS	Analyzes wavelength-dependent surface reflection properties	Provides surface sensitivity but limited morphological discrimination; requires careful calibration for quantitative use.
Raman Spectroscopy	Raman microscopy, confocal imaging	Detects inelastic scattering frequency shifts induced by molecular vibrations	Enables chemical mapping with high specificity, but the inherently weak Raman signals demand long acquisition times.
Fluorescence Spectroscopy	Time-resolved micro-fluorescence	Detects excitation–emission spectra from fluorescent markers	Achieves high sensitivity and selectivity; however, fluorophore tagging is often required and may alter sample properties.
Dispersive Focus Profiling	Chromatic confocal technology (CCT)	Encodes depth information via wavelength-dependent focal shifts	Provides non-contact, tilt-tolerant 3D profiling for complex or transparent surfaces.

## Data Availability

No new data were created or analyzed in this study. Data sharing is not applicable to this article.
